# α-Synuclein aggregation in the olfactory bulb induces olfactory deficits by perturbing granule cells and granular–mitral synaptic transmission

**DOI:** 10.1038/s41531-021-00259-7

**Published:** 2021-12-13

**Authors:** Fengjiao Chen, Wei Liu, Penglai Liu, Zhen Wang, You Zhou, Xingyu Liu, Anan Li

**Affiliations:** 1 Jiangsu Key Laboratory of Brain Disease and Bioinformation, Research Center for Biochemistry and Molecular Biology, Xuzhou Medical University, Xuzhou, China; 2 School of Life Science, Xuzhou Medical University, Xuzhou, China

**Keywords:** Olfactory system, Parkinson's disease

## Abstract

Olfactory dysfunction is an early pre-motor symptom of Parkinson’s disease (PD) but the neural mechanisms underlying this dysfunction remain largely unknown. Aggregation of α-synuclein is observed in the olfactory bulb (OB) during the early stages of PD, indicating a relationship between α-synuclein pathology and hyposmia. Here we investigate whether and how α-synuclein aggregates modulate neural activity in the OB at the single-cell and synaptic levels. We induced α-synuclein aggregation specifically in the OB via overexpression of double-mutant human α-synuclein by an adeno-associated viral (AAV) vector. We found that α-synuclein aggregation in the OB decreased the ability of mice to detect odors and to perceive attractive odors. The spontaneous activity and odor-evoked firing rates of single mitral/tufted cells (M/Ts) were increased by α-synuclein aggregates with the amplitude of odor-evoked high-gamma oscillations increased. Furthermore, the decreased activity in granule cells (GCs) and impaired inhibitory synaptic function were responsible for the observed hyperactivity of M/Ts induced by α-synuclein aggregates. These results provide direct evidences of the role of α-synuclein aggregates on PD-related olfactory dysfunction and reveal the neural circuit mechanisms by which olfaction is modulated by α-synuclein pathology.

## Introduction

Parkinson’s disease (PD) is a neurodegenerative disease characterized by motor deficits, including bradykinesia, rigidity, resting tremor, and postural instability. Those motor deficits are caused by massive loss of dopaminergic neurons in the substantia nigra and the appearance of Lewy bodies in the remaining nigral dopaminergic neurons, leading to functional impairment of the striatum, a structure critical for motor control. Interestingly, a variety of non-motor symptoms are also commonly observed in PD patients, with some of these non-motor symptoms usually preceding the motor symptoms by several years. For instance, olfactory dysfunction is the most prevalent non-motor symptom of PD, presenting in at least 90% of cases, and often precedes motor disturbances by at least 5–10 years^1–3^. Strikingly, although olfactory dysfunction is closely related to PD and is considered a possible biomarker of PD, the neural mechanism underlying this dysfunction remains largely unknown.

A well-known pathologic hallmark of PD is the deposition of abnormal α-synuclein aggregates, the primary component of Lewy bodies, within neurons. It has been indicated that the double mutant α-synuclein (A53T and A30P) has an increased propensity to form aggregates compared with wild-type α-synuclein^4–6^. According to Braak’s staging hypothesis of PD, abnormal α-synuclein deposits follow a specific distribution pattern in the central nervous system, with important olfactory centers such as the olfactory bulb (OB) and the anterior olfactory nucleus being the first sites showing deposits^7–9^. Since abnormal α-synuclein is deposited earlier in the OB than in the substantia nigra, this may at least partly explain why the olfactory dysfunction is observed prior to the motor symptoms in PD. Thus, identifying how abnormal α-synuclein affects the function of the OB and olfaction may provide crucial information for the pathological processes in PD.

The OB is the first processing hub in the olfactory system and is important for the representation of basic initial aspects of olfaction, such as odor identity, intensity, and timing^10^. Although previous studies have reported a relationship between α-synuclein aggregates within the OB and the olfactory perceptual deficits in an animal model of PD^11–14^. They also found that aggregated α-synuclein in the OB can impair olfactory neurogenesis^15^, maturation of newborn neurons^16–18^, homeostasis of the cholinergic and dopaminergic systems in the OB^13^, and neural activity at the network level^19^, very few studies have focused on the mechanisms by which α-synuclein aggregates alter neural activity at the single-cell or synaptic level. This is important because the spikes from single cells and synaptic transmission between different types of cells at the neural network level provide direct information on the functioning of the OB.

To address how α-synuclein directly affects activity in OB neurons and to investigate the underlying neural mechanisms, we induced α-synuclein aggregation almost restrict in the OB by overexpressing double mutant human α-synuclein (A53T and A30P) via targeted injections of an adeno-associated viral (AAV) vector. After observing that α-synuclein aggregates impaired olfactory-related behaviors and altered the normal neural activity of OB neurons in vivo, both in single cells and at the population level, we further demonstrated that the decreased activity in granule cells (GCs) and impaired inhibitory synaptic transmission are likely responsible for the changes in neural activity induced by α-synuclein aggregation in the OB.

## Results

### Specific induction of α-synuclein aggregates in the OB

**Fig. 1:**
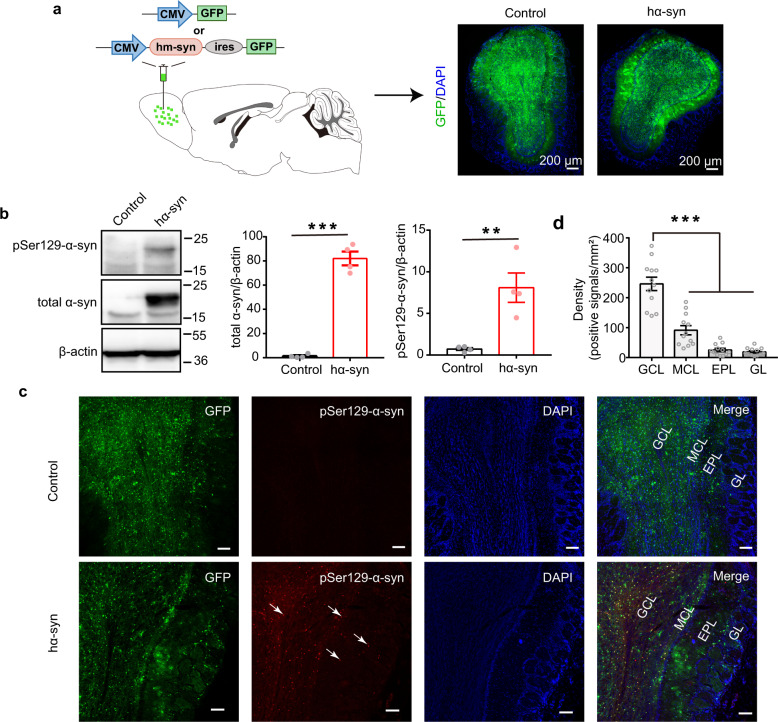
Exogenous high human α-synuclein expression generates aggregates in the OB. **a** Schematic of the viral injections (upper) and the GFP-labeling patterns in the OB of animals injected with AAV-CMV-hm-syn-ires-GFP (hα-syn) or AAV-CMV-GFP (control) (lower). **b** The expression of total α-syn and pSer129-α-syn in OB tissue 3 weeks after viral injection, measured with western blots. *n* = 4 mice for each group. All blots derive from the same experiment and that they were processed in parallel. **c** Representative images showing GFP-positive, pSer129-α-syn-positive, and co-labeled cells in different layers of the OB 3 weeks after viral injection. The white arrows indicate α-syn aggregates. Scale bars = 100 μm. **d** Quantitative analysis of the density of pSer129-α-syn-positive signals in different layers of OB. *n* = 12 slices from 4 mice for each group. GCL granule cell layer, MCL mitral cell layer, EPL external plexiform layer, GL glomerular layer, α-syn α-synuclein; **p < 0.01; ***p < 0.001. Data are presented as mean ± SEM.

To induce α-synuclein pathology specifically in the OB, we injected AAV9-CMV-hm-Syn-ires-GFP into the bilateral OB of C57BL/6J mice to overexpress human mutant α-synuclein and green fluorescent protein (GFP; hα-syn group; Fig. 1a). Control mice received bilateral OB injections of AAV9-CMV-GFP to express GFP only. Transduction efficiency was evaluated by examining the GFP expression pattern with a confocal microscope 3 weeks after the viral injection. Three weeks transduction allowed fully expression of the AAV virus local in the OB. As shown in Fig. 1c, GFP-positive cells were observed in OB slices from both the GFP-control and hα-syn groups. Furthermore, western blot analysis with an anti-α-synuclein antibody showed that the expression of exogenous human total α-synuclein was significantly higher in OB tissue from mice injected with AAV-hα-syn than the tissue from control mice (Fig. 1b and Supplemental Fig. 1a, unpaired *t* test, *t*_(6)_ = 14.11, *p* = 7.92 × 10^−6^). To confirm the occurrence of α-synuclein pathology, the level of pSer129 α-synuclein, a marker of α-synuclein aggregation^4^, was measured in the OB. As expected, the exogenous pSer129 α-synuclein level was significantly higher in the OB of mice injected with AAV-hα-syn than in control mice (Fig. 1b and Supplemental Fig. 1a, unpaired *t* test, *t*_(6)_ = 4.17, *p* = 0.006). In addition, only the mice in the hα-syn group showed strong pSer129 α-synuclein signaling in the OB. Moreover, the pSer129 α-synuclein positive signals were found in almost all layers of the OB, including granule cell layer (GCL), mitral cell layer (MCL), external plexiform layer, and glomerular layer (Fig. 1c). Further analysis showed that the density of pSer129 α-synuclein positive signals in the GCL was significantly higher than that in the other layers (Fig. 1d, Kruskal–Wallis (K-S) test, *p* = 8.88 × 10^−8^, *χ*^2^_(3,44)_ = 35.6, *n* = 12 slices from 4 mice).

Next, we examined whether the abnormal α-synuclein aggregates would induce cell apoptosis in OB. Terminal deoxynucleotidyl transferase-mediated dUTP-fluorescein nick end labeling (TUNEL) staining showed that there were nearly no TUNEL-positive cells in the OB of both control and hα-syn mice (Supplemental Fig. 1b). In addition, we detected whether the α-synuclein aggregates have spread to higher olfactory centers. The data showed that there was no positive pSer129 α-synuclein labeling in piriform cortex and olfactory tubercles in either the control group or the hα-syn group (Supplemental Fig. 2a, b), and only very few α-synuclein aggregates in the anterior part of the anterior olfactory nucleus, which is very close to the OB in hα-syn mice (Supplemental Fig. 2c). Finally, we further confirmed that GFP^+^ cells were not detected in the nasal epithelium (Supplemental Fig. 3), indicating that the virus did not retrogradely transport to upstream olfactory areas. Thus, these results suggest that high expression of human mutant α-synuclein induced α-synuclein pathology. Importantly, the α-synuclein pathology were mainly localized to the OB and rarely spread to higher olfactory centers in our present study (within the timeframe used here).

### Overexpression of human mutant α-synuclein in the OB results in olfactory dysfunction

**Fig. 2:**
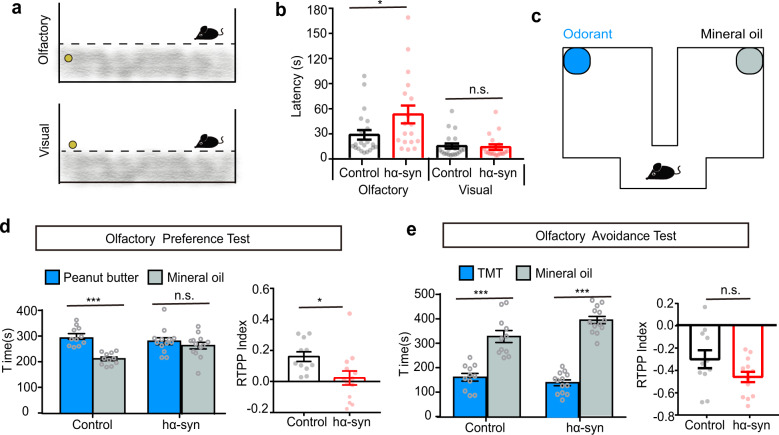
Overexpressing α-synuclein in the OB impairs olfactory function in mice. **a** Schematic of the buried food pellet test. **b** Latency to locate a food pellet that is buried (Olfactory) or on the surface of the bedding (Visual) in control (*n* = 21) and hα-syn (*n* = 18) mice. **c**–**e** Olfactory preference/avoidance test. **c** Schematic of the test chamber. **d** Time spent by control (*n* = 11) and hα-syn mice (*n* = 13) in the chamber with peanut butter (blue) versus mineral oil (green) in the olfactory preference test. **e** Time spent by mice (*n* = 11 for control group and *n* = 13 for hα-syn group mice) in the chamber with TMT (blue) versus mineral oil (green) in the olfactory avoidance test. The RTPP index indicates the degree of preference for peanut butter (**d**) or TMT (**e**). *p < 0.05; ***p < 0.001; n.s. not significant. Data are presented as mean ± SEM.

Although a series of studies in both genetic and drug-induced PD mouse models have shown impairments in olfactory-related behaviors^13,20,21^, it is not clear whether selective induction of α-synuclein aggregation in the OB would influence olfactory perception. Here we directly tested the effect of OB α-synuclein pathology on olfactory function. First, we tested olfactory sensitivity with the buried food pellet test (Fig. 2a). In this commonly used and reliable test, mice use their olfactory sense to locate a buried food pellet^22,23^. Mice overexpressing human mutant α-synuclein took significantly longer time to locate a food pellet buried beneath the bedding than mice in the control group (Fig. 2b, Mann–Whitney *U* test, Mann–Whitney *U* = 112, *p* = 0.029). However, the control and hα-syn mice took a similar length of time to locate a food pellet on the surface of the bedding (Fig. 2b, Mann–Whitney *U* test, Mann–Whitney *U* = 116.7, *p* = 0.55). These results indicate that olfactory ability was affected in hα-syn yet that they have intact motivation to acquire food.

We next asked whether mice in the hα-syn group have deficits in their ability to perceive odors as pleasant or aversive. Using the custom-designed test chamber shown in Fig. 2c, we performed an olfactory preference/avoidance test, which measures how long mice spend investigating filter paper containing an odorant versus mineral oil^24,25^. Peanut butter was used as an attractant and 2,4,5-trimethylthiazole (TMT), a synthetic compound derived from fox feces that induces innate fear and stress responses among rodents, was used as an aversive odorant. In the innate olfactory preference test, mice in the control group spent a significantly higher proportion of time in the chamber with the peanut butter than in the chamber with mineral oil (Fig. 2d, paired *t* test, *t*_(10)_ = 4.90, *p* = 6 × 10^−4^) but mice in the hα-syn group showed no significant difference in time spent in the two chambers (Fig. 2d, paired *t* test, *t*_(12)_ = 0.52, *p* = 0.61). The real time place preference (RTPP) index was significantly lower for hα-syn mice than for control mice (Fig. 2d, unpaired *t* test, *t*_(22)_ = 2.41, *p* = 0.02). In the innate olfactory avoidance test, however, both groups of mice showed significant aversive behavior to TMT (Fig. 2e, paired *t* test, control: *t*_(10)_ = 4.43, *p* = 0.001; hα-syn: *t*_(12)_ = 9.67, *p* < 0.0001), and there was no significant difference between the groups in the observed aversive index (Fig. 2e, unpaired *t* test, *t*_(22)_ = 1.78, *p* = 0.09). Taken together, these results show that human mutant α-synuclein overexpression in the OB leads to a deficit in the innate response to attractive odorants.

### α-Synuclein aggregates enhance both baseline firing and odor-evoked activity in mitral/tufted cells (M/Ts) in the OB

**Fig. 3:**
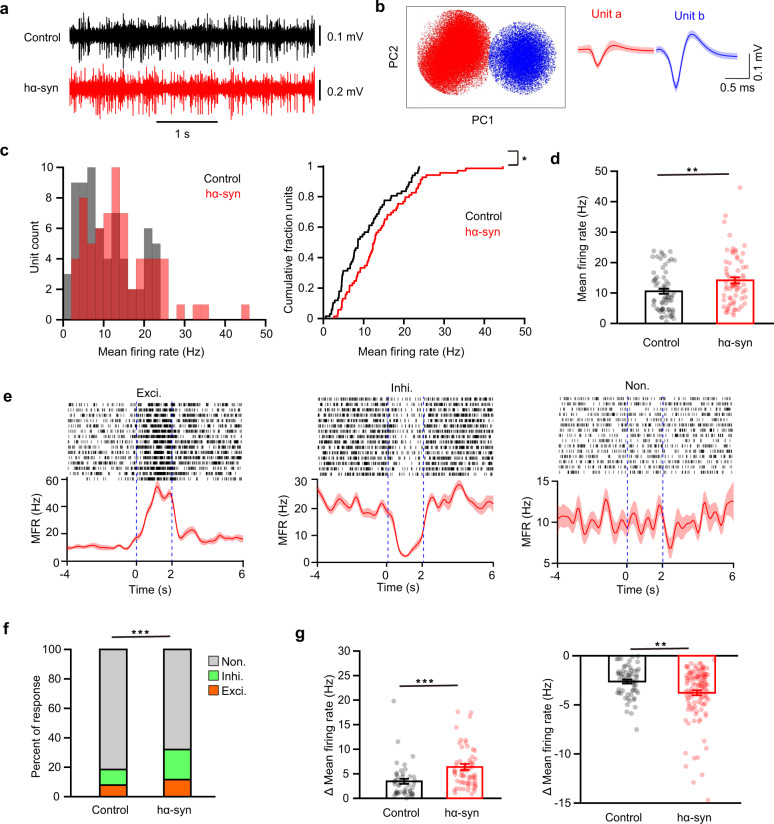
Spontaneous and odor-evoked firing of M/Ts recorded from the OB in vivo. **a** Representative raw spike traces from control and hα-syn mice. **b** Example of spike detection and sorting using PCA clustering of extracellular microelectrode recordings, resulting in separation of two single units (unit a, red; unit b, blue). **c** Histogram (left) and cumulative probability (right) of the baseline mean firing rate (MFR) for all cells recorded. **d** Comparison of the spontaneous firing rate of M/T cells in the OB of control and hα-syn mice. **e** Three examples of cell firing responses to odor presentation. From left to right: an excitatory response, an inhibitory response, and no response. Dotted blue lines indicate the onset and offset of odor presentation. **f** Stacked bar plots showing the percentage of odor-evoked M/T responses in control (*n* = 8) and hα-syn (*n* = 10) mice. **g** Quantification of the change in firing rate of odor-evoked excitatory (left: *n* = 45 and *n* = 64 unit–odor pairs for the control and hα-syn groups, respectively) and inhibitory (right: *n* = 60 and *n* = 113 unit–odor pairs for the control and hα-syn groups, respectively) responses in the two groups. *p < 0.05; **p < 0.01; ***p < 0.001. Data are presented as mean ± SEM.

To investigate the underlying neural basis by which α-synuclein aggregates in the OB impair normal olfactory function, we performed electrophysiological recordings in the OB in awake mice. We recorded spikes from the M/Ts because these are the main output neurons of the OB and play a crucial role in the neural representation of odor information. Individual M/T units were identified by microelectrodes (see “Methods” for details). Although there are many types of cells in the OB, only the spikes from M/Ts could be recorded by array/tetrode recording^26,27^. As in previous studies, strong spontaneous firing of M/Ts was observed (Fig. 3a), and single units could be clearly sorted from the raw spikes (Fig. 3b). A total of 67 and 69 single units were identified in the control and hα-Syn groups, respectively. The distribution of firing rates is shown in Fig. 3c. Compared with the control group, spontaneous firing of M/Ts was significantly greater in the hα-syn group (Fig. 3c, two sample K-S test, *Z* = 0.23, *p* = 0.04 and Fig. 3d, Wilcoxon rank sum test, rank sum = 3989, *p* = 0.009). Thus, aggregation of α-synuclein in the OB enhances the ongoing baseline activity of M/Ts.

Next, we investigated whether aggregation of α-synuclein in the OB affected odor-evoked responses in M/Ts. We recorded the response of M/Ts to presentation of eight different odors (Fig. 3e). As in previous studies, many units showed no significant response to odors, but some units showed excitatory or inhibitory responses (Fig. 3e). Compared with control mice, hα-syn mice had a significantly higher percentage of cells with an odor-evoked response (Fig. 3f, Pearson Chi-Square test, *χ*^2^_(2)_ = 28.57, *p* = 6.3 × 10^−7^). Strikingly, the percentage of cells with inhibitory response was increased nearly 2-fold (11 vs 20%) and that of the cells with excitatory response was increased 1.5-fold (8 vs 12%, Fig. 3f). Furthermore, responsive cells in the hα-syn group showed significantly larger changes in firing for both excitatory and inhibitory responses than cells in the control group (Fig. 3g, Wilcoxon rank sum test, rank sum = 1794*, p* = 2.8 × 10^−5^ for excitatory responses; rank sum = 6050*, p* = 0.008 for inhibitory responses). Together, these results from single-unit recordings indicate that α-synuclein pathology in the OB enhances both spontaneous firing and odor-evoked responses in M/Ts, resulting in abnormal hyperactivity of the M/Ts in the OB.

### α-Synuclein aggregates increase odor-evoked high-gamma oscillations in the OB

**Fig. 4:**
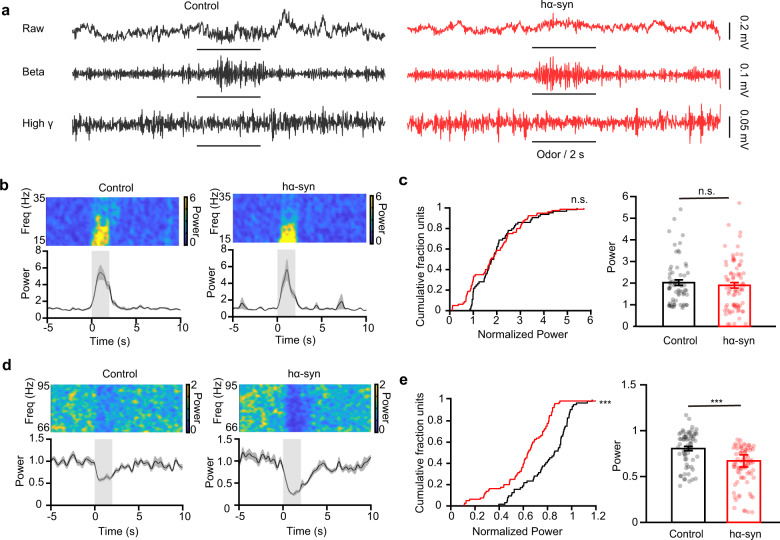
α-Synuclein aggregates change the odor-evoked high-gamma response in the OB. **a** Examples of raw traces (top) and filtered beta (middle) and high-gamma (bottom) oscillations in the LFP signals recorded from representative control and hα-syn mice in vivo with odor stimulation. **b**, **d** Example power spectra (top) and trial-averaged normalized traces (bottom) of beta (**b**) and high-gamma (**d**) responses elicited by isoamyl acetate delivery (gray boxes). **c**, **e** Power in the normalized odor-evoked beta band (**c**) and high-gamma band (**d**) in the two groups (odor–mouse pair: *n* = 64 for the control group; *n* = 80 for the hα-syn group). ***p < 0.001; n.s. not significant. Data are presented as mean ± SEM.

Whereas single-unit spikes reflect the neural activity from an individual cell, oscillations in the local field potential (LFP) reflect the network activity from a population of different types of closely related neurons and connected input circuits. Different oscillation frequencies in the OB LFP carry important information about the chemical properties of odors, odor discrimination, and olfactory learning. Thus, to investigate how α-synuclein pathology affects LFP oscillations in the OB, we recorded ongoing and odor-evoked LFPs in the OB of awake mice (Supplemental Fig. 4a and Fig. 4a). We focused our analysis on the beta (15–35 Hz) and high-gamma (66–95 Hz) oscillations since they reflect circuit processing for odor detection and discrimination and show strong and reliable responses to odor stimulation. We found that α-synuclein aggregation had no significant effect on either beta or high-gamma oscillations in the ongoing LFP recorded from the OB (Supplemental Fig. 4, two-sample *t* test, *t*_(16)_ = 1.35, *p* = 0.20 (beta); *t*_(16)_ = 0.56, *p* = 0.58 (high gamma)). We next investigated the difference between odor-evoked responses recorded from control mice and hα-syn mice. Figure 4a shows example LFP traces in response to isoamyl acetate presentation in a control mouse and a hα-syn mouse, with strong increases in the beta oscillation and clear decreases in the high-gamma oscillation in both mice (Fig. 4a). Although the amplitude of the odor-induced beta oscillation response was similar in the representative hα-syn mouse and the representative control mouse (Fig. 4b), the amplitude of the odor-induced high-gamma oscillation response was clearly greater in the hα-syn mouse (Fig. 4d). When we compared the responses across different mice and odors, we found that α-synuclein aggregation did not affect the amplitude of the odor-evoked beta response (Fig. 4c, left: two-sample K-S test, *Z* = 0.20, *p* = 0.09; right: Wilcoxon rank sum test, rank sum = 4840, *p* = 0.42) but did significantly increase the extent of the odor-evoked high-gamma response (Fig. 4e, left: two-sample K-S test, *z* = 0.46, *p* = 3.72 × 10^−7^; right: Wilcoxon rank sum test, rank sum = 5915, *p* = 2.99 × 10^−7^). Thus, α-synuclein aggregation changes odor-evoked high-gamma oscillations in the OB.

### Hyperactivity in mitral cells induced by α-synuclein aggregation is due to weakened inhibitory input

**Fig. 5:**
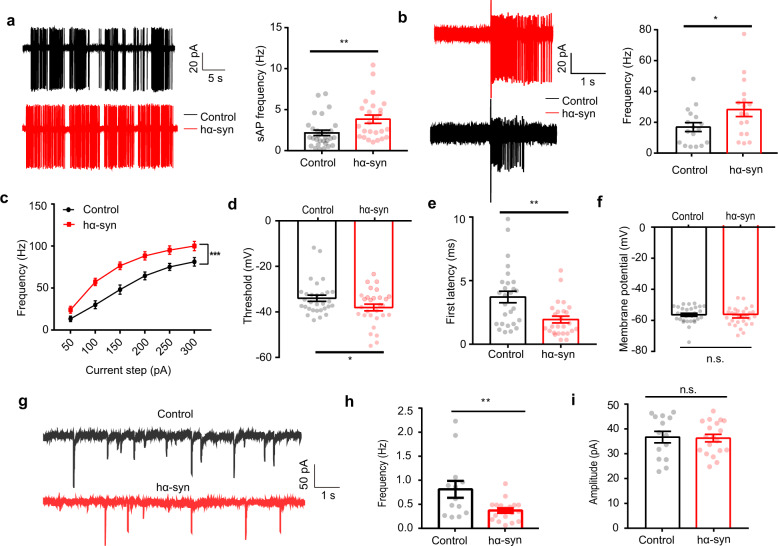
α-Synuclein aggregates increase spontaneous and evoked firing in mitral cells in vitro. **a** Representative (left) and quantitative analysis (right) of spontaneous firing rates of mitral cells (control: 30 cells from 13 mice; hα-syn: 25 cells from 15 mice) over a period of 5 min. **b** Representative (left) and quantitative analysis (right) of olfactory-nerve-evoked firing rates of mitral cells (17 cells from 11 control mice and 17 cells from 8 hα-syn group mice) in a 1.8 s recording period (olfactory nerve stimulation; 200 ms, 400 mA). **c**–**f** Quantitative analysis of the frequency (**c**), threshold voltage to trigger first AP (**d**), onset latency (**e**), and membrane potential (**f**) elicited by positive current injections (control: 30 cells from 11 mice; hα-syn: 29 cells from 7 mice). **g** Representative mIPSCs recorded from M/Ts in control and hα-syn slices. **h**, **i** Quantitative analyses of mIPSCs frequency (**h**) and amplitude (**i**) over a 5-min recording period (*n* = 14 neurons from 5 mice and 19 neurons from 4 mice; control and hα-syn groups, respectively). sAP spontaneous action potential, mIPSC miniature inhibitory postsynaptic current; *p < 0.05; **p < 0.01; ***p < 0.001; n.s. not significant. Data are presented as mean ± SEM.

Since α-synuclein aggregation in the OB leads to hyperactivity in single M/Ts in vivo, we next asked how aggregated α-synuclein leads to these changes in neural activity. To study the underlying cellular and synaptic mechanisms, we performed slice recordings in vitro. In the cell-attached configuration, mitral cells showed strong spontaneous firing in both control slices and slices from hα-syn mice (Fig. 5a). Consistent with the findings in single-unit recordings in awake mice, spontaneous firing rates of mitral cells were significantly higher in the OB slices from hα-syn mice than in the control slices (Fig. 5a, Mann–Whitney *U* test, Mann–Whitney *U* = 206, *p* = 0.004). To mimic odor-evoked mitral cell responses in vitro, we recorded mitral cell activity during electrical stimulation of the axon terminals from olfactory sensory neurons (Fig. 5b). We found that the firing rate of mitral cells during electrical stimulation was significantly higher in hα-syn slices than in control slices (Fig. 5b, unpaired *t* test, *t*_(32)_ = 2.11, *p* = 0.04).

To further test whether α-synuclein aggregation in the OB affects mitral cell excitability, we recorded current-evoked action potentials (APs) from mitral cells in current-clamp mode. APs were elicited by positive current injection with current intensities ranging from 50 to 300 pA; the frequency of APs increased with the size of the current step in both control and hα-syn slices (Supplemental Fig. 5a). Compared with control slices, cells from hα-syn slices showed significantly higher firing rates (Fig. 5c, two-way analysis of variance (ANOVA), *F*_(1, 57)_ = 14.07, *p* = 4×10^−4^), lower thresholds (Fig. 5d, unpaired *t* test, *t*_(57)_ = 2.04, *p* = 0.046), and shorter onset latencies (Fig. 5e, unpaired *t* test, *t*_(51)_ = 3.33, *p* = 0.002) for the evoked spikes, but the membrane potential remained unchanged (Fig. 5f, unpaired *t* test, *t*_(57)_ = 0.7027, *p* = 0.485). These results further confirm the observation that α-synuclein aggregation increases the excitability of mitral cells.

In the OB, mitral cells receive intensive inputs from GABAergic interneurons. The increased excitability of mitral cells caused by aggregation of α-synuclein observed above might be a direct effect of α-synuclein on the mitral cells or an indirect effect via the GABAergic interneurons. To test whether inhibitory inputs are involved in the effect of α-synuclein, we recorded miniature inhibitory postsynaptic currents (mIPSCs) in mitral cells (Fig. 5g). We found that α-synuclein aggregation significantly reduced the frequency (Fig. 5h, unpaired *t* test, *t*_(30)_ = 2.83, *p* = 0.008) but not the amplitude of mIPSCs (Fig. 5i, unpaired *t* test, *t*_(31)_ = 0.15, *p* = 0.88). These results indicate that the inhibitory inputs to mitral cells are weakened by α-synuclein aggregation in the OB.

### GCs play an important role in the M/T hyperactivity induced by α-synuclein aggregates in the OB

**Fig. 6:**
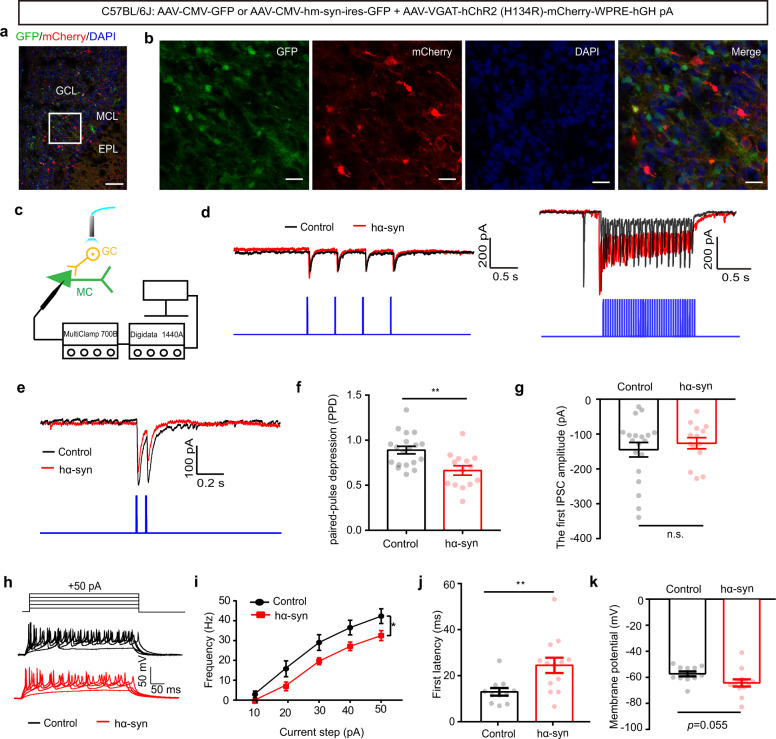
α-Synuclein aggregates reduce paired-pulse depression of light-evoked IPSCs recorded from mitral cells and decrease granule cell activity in the OB. **a**, **b** Low-magnification (scale bars = 100 μm) (**a**) and high-magnification (scale bars = 20 μm) (**b**) images showing granule cells labeled with mCherry in the OB. EPL external plexiform layer, MCL mitral cell layer, GCL granule cell layer. **c** Schematic of the light-evoked IPSC electrophysiological recording. **d** Representative IPSCs recorded from mitral cells when 2 Hz (left) and 20 Hz (right) light illumination was applied. **e** Representative traces of paired-pulse IPSCs. **f** Quantitative analysis of the paired-pulse depression of light-evoked IPSCs recorded from mitral cells from the control and hα-syn groups (control: 19 cells from 8 mice; hα-syn: 14 cells from 6 mice). **g** Quantitative analysis of the amplitude of the first light-evoked IPSC. **h** Representative current injection-evoked APs in granule cells. **i**–**k** Quantitative analysis of the frequency (**i**), onset latency (**j**), and membrane potential (**k**) elicited by positive current injections (13 cells from 5 mice for the control and hα-syn groups, respectively). mIPSC miniature inhibitory postsynaptic current; *p < 0.05; **p < 0.01; n.s. not significant. Data are presented as mean ± SEM.

Although several classes of GABAergic interneuron send direct inhibitory inputs to mitral cells in the OB, GCs are the major class and dramatically modulate mitral cell activity via dendro-dendritic synapses. Thus, we next set out to test whether α-synuclein aggregation affects inhibitory transmission from GCs to mitral cells. We selectively delivered channelrhodopsin-2 (ChR2) to GCs via injection of AAV2/9-VGAT-hChR2-mCherry. Three weeks after viral injection, we observed mCherry was expressed restricted to GCs (Fig. 6a, b). This was further confirmed by immunofluorescence (Supplemental Fig. 6a). To assess the properties of the photocurrents mediated by activation of ChR2 in neurons, we performed whole-cell recordings from GCs in acute slices with light stimulation (Supplemental Fig. 5b). We found that 2 Hz photostimulation with a 473-nm laser evoked inward currents in voltage-clamp mode (Supplemental Fig. 5c) and faithfully evoked APs in current-clamp mode (Supplemental Fig. 5d). Under the same conditions, we recorded light-evoked IPSCs (eIPSCs) in mitral cells when GCs in the slice were optogenetically simulated (Fig. 6c). As expected, both 2 and 20 Hz photostimulation induced stimulus-locked IPSCs in the mitral cells (Fig. 6d). When photostimulation was delivered at an inter-stimulus interval of 80 ms, the paired IPSCs exhibited paired-pulse depression (PPD) (Fig. 6e). As shown in Fig. 6f, g, PPD was significantly lower in the hα-syn group than in the control group (Fig. 6f, unpaired *t* test, *t*_(30)_ = 3.4, *p* = 0.002) but there was no difference in the amplitude of the first eIPSC between the two groups (Fig. 6g, unpaired *t* test, *t*_(31)_ = 0.67, *p* = 0.51). Since changes in the PPD of GABAergic IPSCs indicate a likely presynaptic origin for modulatory effects^28,29^, our results suggest that the effects of α-synuclein aggregates on GABAergic inhibition in the OB may have a presynaptic locus in GCs.

To further confirm that α-synuclein aggregation affects neural activity in GCs, we tested whether the excitability of GCs was altered by α-synuclein overexpression. We recorded current-evoked APs from GCs during different amplitudes of current injection in current-clamp mode (Fig. 6h). Compared with the control group, evoked spikes from cells in the hα-syn group had significantly lower firing rates (Fig. 6i, two-way ANOVA, *F*_(1, 22)_ = 5.88, *p* = 0.024) and longer onset latencies (Fig. 6j, unpaired *t* test, *t*_(22)_ = 2.935, *p* = 0.008), as well as a slight decrease in membrane potential (Fig. 6k, unpaired *t* test, *t*_(22)_ = 2.024, *p* = 0.055). These results indicate that α-synuclein aggregation decreases excitation in GCs.

**Fig. 7:**
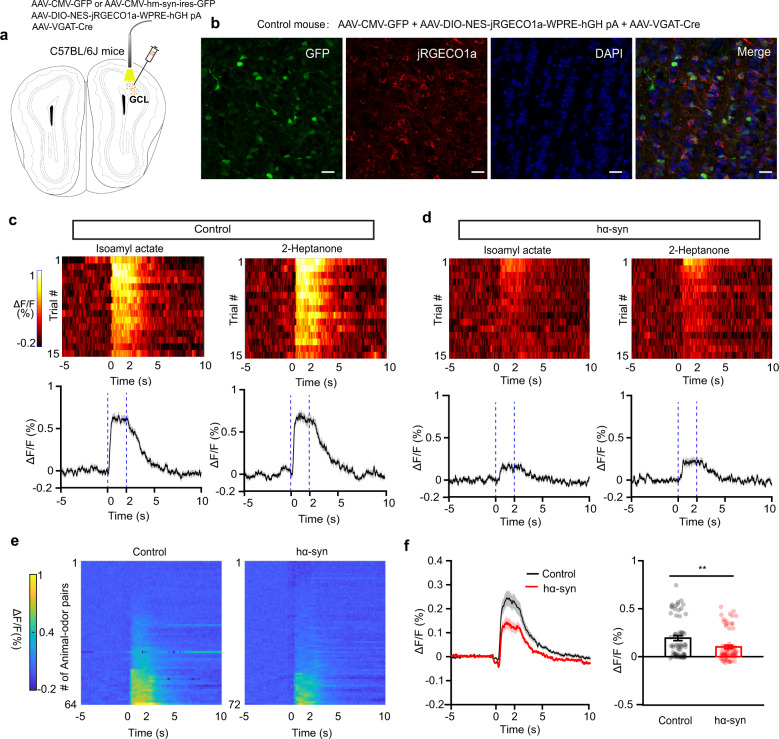
α-Synuclein aggregates impair the odor-evoked calcium responses of granule cells in vivo. **a** Schematic for fiber photometry recording of calcium signals from granule cells expressing jRGECD1a in control or hα-syn mice. **b** Expression of jRGECD1a in the granule cell layer of the OB from a representative control mouse. Scale bars = 20 μm. **c**, **d** Trial-to-trial pseudo-colored heat maps (top) and trial-averaged traces (bottom; mean ± SEM) of the calcium responses evoked by passive exposure to two different odors, recorded from control (**c**) and hα-syn (**d**) mice. **e** Heat map illustrating the calcium responses for all animal–odor pairs recorded from control and hα-syn mice. **f** The averaged calcium response (Δ*F*/*F*, %) of granule cells during odor delivery (*n* = 64 and *n* = 72 animal–odor pairs; control and hα-syn groups, respectively). **p < 0.01. Data are presented as mean ± SEM.

Finally, to confirm that α-synuclein aggregation affects neural function in GCs, we performed in vivo recordings in awake mice. Since it is difficult to record GC spikes extracellularly, we instead recorded the population calcium activity in GCs, which indirectly reflects neural activity. We recorded the real-time calcium dynamics in GCs in head-fixed awake mice during odor presentation via a fiber photometry system. Three weeks before recording, we injected a mixture of AAVs (AAV-CMV-GFP, AAV-DIO-NES-jRGECO1a-WPRE-hHG pA, and AAV-VGAT-Cre for control mice and AAV-CMV-hm-Syn-ires-GFP, AAV-DIO-NES-jRGECO1a-WPRE-hHG pA, and AAV-VGAT-Cre for hα-syn mice) into the GCL to induce specific expression of a red calcium indicator, jRGECO1a, in GCs (Fig. 7a, b). The expression specificity of the AAV-DIO-NES-jRGECO1a-WPRE-hHG pA was verified by immunofluorescence experiment (Supplemental Fig. 6b). Odor presentation could evoke a strong increase in the population calcium signal in GCs in both control and hα-syn mice (Fig. 7c, d). Since calcium activity recorded by fiber photometry is a relative value and is usually used to evaluate event-related activity rather than ongoing spontaneous activity, we analyzed only the odor-evoked responses. We found that, while odors evoked strong responses from most animals in the control group, they evoked only weak response in the hα-syn group (Fig. 7e). In general, the odor-evoked calcium response in GCs was significantly reduced in the hα-syn group compared with the control group (Fig. 7f, Wilcoxon rank sum test, rank sum = 5040, *p* = 4.26 × 10^−3^). These results indicate that α-synuclein aggregates in the OB decrease odor-evoked neural activity in GCs. Therefore, GCs appear to be critically involved in the hyperactivity of M/Ts observed above.

### α-Synuclein aggregates alter the presynaptic structure of GCs in the OB

**Fig. 8:**
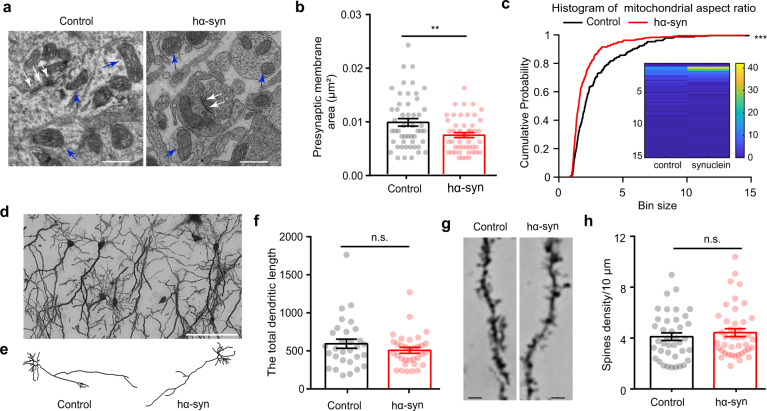
Morphological changes induced by α-synuclein aggregation in the olfactory bulb. **a** Representative electron micrograph showing the synaptic ultrastructure (white arrows) and mitochondria (blue arrows) in neurons. Scale bars = 500 nm. **b** Quantitative analysis of the area of the presynaptic membrane in the two groups of mice (control: *n* = 51 synapses from 4 mice; hα-syn: *n* = 49 synapses from 4 mice). **c** Quantitative analysis of the mitochondrial aspect ratio (control: *n* = 231 mitochondria from 4 mice; hα-syn: *n* = 343 mitochondria from 4 mice). **d** Representative Golgi staining photomicrograph showing neurons in the granule cell layer of the OB. Scale bars = 20 μm. **e** Representative reconstructions of granule cells from the OB of mice in the two groups. **f** Comparison of the total dendritic length of granule cells in the two groups of mice (control: *n* = 29 cells from 4 mice; hα-syn: *n* = 32 cells from 4 mice). **g** Representative dendrites and spines of granule cells obtained from control and hα-syn mice. Scale bars = 2 μm. **h** Spine density on granule-cell dendrites (control: *n* = 39 dendrites from 3 mice; hα-syn: n = 42 dendrites from 3 mice). **p < 0.01; ***p < 0.001; n.s. not significant. Data are presented as mean ± SEM.

It has been reported that abnormal α-synuclein accumulation occurs mainly in the presynaptic terminals of neurons in the brain^30^; thus, it is likely that the weakened synaptic transmission from GCs to mitral cells observed in hα-syn mice is due to the changes in presynaptic structure in GCs. To test this hypothesis, we measured the presynaptic structure and mitochondria using electron microscopy (Fig. 8a). We found that the presynaptic membrane area was smaller in the hα-syn group than in the control group (Fig. 8b, unpaired *t* test, *t*_(97)_ = 2.819, *p* = 0.0058). In addition, the mitochondria observed in the synaptic area in the hα-syn group had a thicker and shorter phenotype than the control group, as reflected in the mitochondrial aspect ratio index (Fig. 8a (blue arrows) and Fig. 8c, two-sample K-S test, *Z* = 0.26, *p* = 6.49 × 10^−9^).

Lastly, we examined the dendritic processes and spines of GCs using Golgi staining. Figure 8d shows a representative photomicrograph of GCs from the granular cell layer of the OB, and Fig. 8e shows two representative reconstructions of GCs from hα-syn and control mice. We found no significant difference in the total dendritic length for GCs from control and hα-syn mice (Fig. 8f, unpaired *t* test, *t*_(58)_ = 1.3, *p* = 0.1985). Furthermore, the density of dendritic spines on the GCs was not changed by α-synuclein aggregates (Fig. 8g, h, unpaired *t* test, *t*_(78)_ = 0.744, *p* = 0.0.459). Taken together, the morphological results suggest that α-synuclein aggregates alter the presynaptic structure, but not the dendritic complexity, of GCs in the OB.

## Discussion

In this study, we investigated how α-synuclein aggregation in the OB affects olfactory behaviors and neuronal activity at the cellular and synaptic levels. Our data demonstrate that specific overexpression of α-synuclein aggregates in the OB results in deficits in olfactory detection and in the innate responses to attractive odors in mice (Figs. 1 and 2). This olfactory dysfunction is likely due to functional changes in neural activity in the OB, particularly hyperactivity of M/Ts in response to odors (Figs. 3 and 4). Moreover, data from in vitro slice recordings and morphological analysis of GCs indicate that this mitral cell hyperactivity is the result of weakened inhibitory input from GCs and synaptic dysfunction. These findings provide direct evidences for the deleterious effects of α-synuclein aggregation in the OB on the normal functioning of OB neurons and identify the cellular mechanisms.

Since abnormal aggregation of α-synuclein in the olfactory system is a critical feature of early-stage PD, several previous studies have investigated its effects on olfactory function in rodents and patients. Using a transgenic mouse model of PD expressing human wild-type α-synuclein under the control of the mouse α-synuclein promoter, Petit et al. found that α-synuclein mice were progressively impaired in their ability to detect odors and to discriminate between odors and exhibited alterations in short-term olfactory memory^12^. Compared with their wild-type littermates, α-Syn^A53T^ transgenic mice aged ≥6 months displayed deficits in odor discrimination and odor detection but no deficits in olfactory memory or odor habituation^13^. In addition, an α-synuclein BAC transgenic mouse model, which nicely recapitulates the native α-synuclein pathology pattern for PD, also exhibited hyposmia and rapid eye movement sleep behavior disorders but without motor symptoms^31^. In humans, the Lewy body pathology was detected in the olfactory epithelium in six of the eight patients with PD, which indicated the close relationship of α-synuclein pathology and olfaction function^32^.

Since the OB is the first olfactory center in the brain and one of the earliest sites for α-synuclein aggregation, olfactory dysfunction after α-synuclein aggregation is commonly explained as an impairment in the OB. However, many of the previous studies were performed in α-synuclein transgenic mice or in PD patients with abnormal α-synuclein aggregation distributed across many brain areas, rather than being restricted to the OB. This raises the question of whether aggregation of α-synuclein in the OB alone would also result in olfactory dysfunction. A recent study reported that rats overexpressing human mutant (A53T and A30P) α-synuclein in the OB after AAV injection exhibited a progressive impairment in odor discrimination from 3 to 12 week after virus injection^4^. We further answer this question directly in the present study by showing that olfactory detection is decreased after α-synuclein overexpression in the OB in mice.

M/Ts are the major output neurons of the OB: they receive direct excitatory input from the olfactory sensory neurons^33^ and send the neural information processed by the circuits within the OB to higher olfactory centers. Electrophysiological signals recorded from M/Ts play important roles in the discrimination of odors and olfactory-related learning. While spikes from single M/Ts can carry critical information about both odor identity and odor value, LFP signals, especially in the high-gamma band, are critically involved in learning olfactory discriminations. The dysfunction in olfactory behavior can be explained by the fact that neural signals recorded from the OB, including both spikes and odor-evoked high-gamma oscillations, are disturbed by α-synuclein aggregation.

Interestingly, a recent study extensively investigated the changes of LFP signals in the OB and piriform cortex after seeding with the α-synuclein preformed fibrils (PFFs). They found that, while spontaneous activity of beta oscillations remained unchanged, the odor-evoked beta oscillations were significantly elevated in the OB^19^. However, in our study, α-synuclein aggregation disturbed only the odor-evoked high-gamma oscillations with the beta oscillations unchanged. This discrepancy is likely because the α-synuclein PFFs may have spread out of the OB to other olfactory centers, such as the piriform cortex^19^. Whereas in our study, the α-synuclein aggregation was restricted to the OB. In addition, the methods of the induction for the α-synuclein pathology might also contribute this discrepancy: AAV expression within the cells in present study and directly seeding PFFs into the OB in prior study. These two methods may cause the different expression levels and expression bias in cells.

We reported hyperactivity in OB neurons in a previous study in which we established a pre-PD model with olfactory dysfunction^23^. Thus, it was not unexpected that α-synuclein aggregation in the OB would cause hyperactivity in M/Ts, both in spontaneous firing and in the odor-evoked responses. Indeed, it has been widely reported that abnormal protein aggregation can exert a neuro-toxic effect by increasing neuronal excitation. For example, in both cortex and hippocampus, Aβ deposition is positively associated with neuronal excitability^34,35^. Interestingly, Aβ deposition in the OB results in hyperactivity in mitral cells^36^, increased odor-evoked activity in the piriform cortex, and increased OB–piriform cortex functional connectivity^37^. These findings are consistent with the results of the present study showing that α-synuclein aggregation increased the excitability of mitral cells. Thus, Aβ aggregation and α-synuclein aggregation may exert their neuro-toxicity on M/Ts in the OB and on the principal neurons in other brain areas such as the hippocampus and nigra substance via the same mechanism. Although the mechanism of how the increased excitability of M/Ts caused by α-synuclein aggregation leads to olfactory dysfunction is still unclear, it is likely that the excitatory/inhibitory (E/I) balance is critically involved. Excitatory and inhibitory neurons together establish an E/I balance that is essential for normal brain development and function. For example, it is reported that the E/I imbalance widely affects neural computation, in turn giving rise to the broad behavioral symptoms recognized as autism^38^. In the OB, the hyperactivity of M/Ts could disrupt the homeostasis of E/I balance, which is likely the pathogenetic mechanism of hyposmia. Indeed, this hypothesis has been supported by a recent study performed with slice recordings in which the mitral cells in OB burdened with trimeric Aβ showed abnormal hyperactivity and significantly changed E/I balance^39^.

GCs are the predominant class of GABAergic interneurons in the OB and form reciprocal dendro-dendritic synapses with the lateral dendrites of the M/Ts^40^. Therefore, the neural activity and normal function of the M/Ts are heavily modulated by and dependent on the neural activity in the GCs and the synaptic transmission between GCs and mitral cells. Here we provide direct evidence that α-synuclein affects the functioning of M/Ts at least in part via effects on the GCs, which greatly outnumber the M/Ts.

In addition to the hypoactivity of GCs, impairments in synaptic transmission are also responsible for changes in GABA-dependent neuronal inhibition. It has been reported that α-synuclein accumulation reduces GABAergic inhibitory transmission in a model of multiple system atrophy^41^. Consistent with our findings, a recent study found that α-synuclein aggregation induced a reduction in presynaptic membrane area^5^, likely due to binding of α-synuclein aggregates with microtubule beta-III to form an insoluble complex. This complex may also regulate release of synaptic vesicles in GABAergic interneurons^41^.

Studies have reported the great toxic effects of diverse α-synuclein aggregates on neurons, including altering membrane permeability^42,43^, damaging mitochondria^44,45^, triggering lysosomal leakage^46^, disrupting microtubules^47^, or destroying synapses functions^41,48,49^. Moreover, α-synuclein oligomers can block Ca^2+^-dependent inactivation of IP3R, triggering Ca^2+^-induced Ca^2+^ release from intracellular stores and resulting in intraneural Ca^2+^ dyshomeostasis^50^. Since increasing evidence suggests that interneuron populations in the olfactory system are selectively vulnerable to Alzheimer’s disease-related neuropathology^36,51^, similar mechanisms might also be found for synucleinopathy in the OB. That is likely the reason why we found the higher density of α-synuclein aggregates in the GCL. However, the underlying molecular mechanism by which α-synuclein affects GCs in the OB is likely very complex and will require future study.

The OB receives intensive and extensive feedback and modulatory inputs from other higher brain centers, such as piriform cortex, anterior olfactory nucleus, and modulatory system, including serotonergic inputs from dorsal raphe, cholinergic inputs from forebrain, noradrenergic inputs from the locus coeruleus, and even dopaminergic inputs from the substantia nigra. These innervations strongly modulate the neural activity and function in the OB^10,52–55^. The α-synuclein overexpressed in the OB affect the neurons within the OB, and they also would affect these innervations. Therefore, although the α-synuclein aggregates in the OB mainly disrupt the normal function of the local neural circuit in the OB, impairment of centrifugal projections to the OB might be also involved. Since most of these innervations directly enhance the activity of GCs and finally cause inhibitory effect on the mitral cells^53,54^, disruption of them might lead to the hyperactivity of mitral cells which is consistent with the results observed in present study. In addition, although evidence from present study indicates that the overexpression of α-synuclein in the OB affects the neural activity and odor representation of M/Ts mainly via indirect effects by modulating GCs, these aggregates would also directly affect the neural activity and function of M/Ts. Therefore, the α-synuclein overexpression affects the function of M/Ts mainly via effects on the GCs. Other factors, such as the direct effect on the M/Ts and the indirect centrifugal innervations are also likely critically involved.

Olfactory dysfunction is the one of the earliest and most prevalent non-motor symptoms of PD; however, the underling mechanism remains largely unknown. Braak et al. proposed a neuropathological staging procedure in which the pathological process progresses are in six stages. Even in the stage 1, the inclusion body pathology caused by α-synuclein aggregates initially occurs in the OB^7^. Although it is widely accepted that α-synuclein aggregates in the central olfactory system would impair olfactory function, it is still not clear whether α-synuclein aggregates mainly in the OB but not in higher olfactory centers would lead to olfactory dysfunction. Our present study answered this question directly by overexpression of α-synuclein in the OB. Thus, it is very likely that, at very early stage of PD (such as stage 1), olfactory dysfunction is only due to the impairment of the OB caused by α-synuclein aggregates. With the development of the pathological process, higher olfactory centers are involved since the α-synuclein aggregates spread to these brain areas.

In summary, the present study identifies how does the α-synuclein aggregation in the OB impairs spontaneous activity and odor-evoked responses in the OB output neurons, ultimately leading to olfactory dysfunction. These findings are important for understanding the neural mechanisms underlying the toxic effects of α-synuclein on the whole brain and the neural pathology of PD.

## Methods

### Animals

Mice were housed, maintained, and used in experiments under the regulations, approval, and animal care standards of the Institutional Animal Care and Use Committee of Xuzhou Medical University. Adult male C57BL/6J mice were group housed on a reverse light cycle until they underwent surgery, after which mice were housed individually for at least 1 week for recovery before further experiments.

### Overexpression of human mutant α-synuclein in the OB

All the procedures below used mice that received OB injections of either AAV9-CMV-hm-Syn-ires-GFP (7.38 × 10^13^ vg/ml), to overexpress the double-point mutation of human α-synuclein (A30P and A53T) and GFP (hα-syn group), or AAV9-CMV-GFP (5.01 × 10^13^ vg/ml), to express GFP only (control group). Both viruses were purchased from Vigene Biosciences Co. (Shandong, China).

OB viral injections were performed under general anesthesia with pentobarbital sodium (90 mg/kg body weight, intraperitoneal (i.p.)) while animals were mounted on a stereotactic instrument. Mice were fixed on the stereotactic instrument and then AAV9-CMV-hm-Syn-ires-GFP or AAV9-CMV-GFP (350 nl per side) was injected into bilateral OB (anterior–posterior (AP), 4.5 mm; medial–lateral (ML), ±0.65 mm; dorso–ventral (DV), 2.7 mm) at a slow rate (30 nl/min) using a syringe pump. The injection needle was withdrawn 10 min after infusion and the skin was sutured. Mice were returned to their home cage for recovery. To ensure viral expression, there was a waiting period of about 3 weeks after injection before further experiments were performed.

### Olfactory preference/avoidance test

The olfactory preference/avoidance test was conducted as described previously^56^. A custom-designed test chamber (45 cm × 35 cm × 25 cm) with two equally sized compartments was used to determine which odorants were perceived as an attractant or repellent for two groups of mice. In this study, peanut butter was used as the attractant odorant and TMT, a synthetic compound derived from fox feces that induces innate fear and stress responses among rodents^57^, was used to test innate olfactory avoidance behavior. Before testing, mice were placed into the chamber and allowed a 10 min acclimation period. Then 1 g peanut butter was placed in one compartment and 50 μl of the neutral scent (mineral oil) was placed in the other compartment. Mice were exposed to the two odorants for 10 min. Exploratory behavior was recorded by a video camera and the time spent in each chamber was calculated automatically by a computerized recording system. The avoidance test was performed in the same manner by applying 50 μl of TMT (dissolved in mineral oil at 40% v/v dilution) and 50 μl of mineral oil. To analyze the olfactory preference/avoidance data, the test cage was divided into two compartments of equal area. The time that mice spent in each compartment during the 10-min test was measured from the recorded videos using MATLAB. The time spent in the odorant side and in the mineral oil side were compared with a paired *t* test for both the control mice and the hα-syn mice. The RTPP indices of the two groups were compared with an unpaired *t* test. The RTPP index was calculated by the following formula: (time spent in the odorant side − time spent in the mineral oil side)/(time spent in the odorant side + time spent in the mineral oil side).

### Buried food pellet test

The well-established buried food pellet test was used to evaluate odor detection ability. Mice were deprived of food for 24 h before the experiment and during the experiment but had free access to water. Before a single trial, the mouse was placed into a clean test cage for 10 min to habituate to the environment. Then the mouse was taken out and a 200-mg food pellet was buried approximately 0.5 cm below the surface of 3-cm-deep bedding. The mouse was placed into the cage again and was given 300 s to locate the buried pellet. The location of the food pellet was changed at random.

The latency to locate the food pellet was defined as the time between placement of the mouse in the cage and when it touched the food pellet with its forepaws or nose. Mice were allowed to consume the pellet and were then returned to their home cage. As a control, a visible pellet test was carried out the next day: conditions were identical except that the food pellet was placed randomly on top of the bedding.

### Surgery for microelectrode implantation

As previously described, microelectrodes (16 single nichrome wires, single‐wire diameter is 30 μm, the distance between wires is 250 μm, Jiangsu Brain Medical Technology Co. Ltd) were implanted into the OB to record spikes and LFPs. Briefly, mice were anesthetized with pentobarbital sodium (90 mg/kg body weight, i.p.) and positioned in a stereotaxic frame. Eye ointment was applied to the eyes. The fur on the surface of the scalp from the midline of the orbits to the midpoint between the ears was removed. A suitably sized hole was drilled above the right OB for microelectrode implantation (AP, 4.0 mm; ML, 1.0). Then, the microelectrodes were positioned and lowered through the drilled hole to an average depth of 1.8–2.0 mm, targeting the ventral MCL of the OB^56,58–60^. Recordings were made during microelectrode implantation to ensure optimal placement. Two small screws inserted into the skull served as the reference electrode and were connected to the ground. To fix the head during recording, a custom-designed head plate was attached to the skull using dental acrylic, which was also applied to seal the microelectrodes to the bone.

### Spike/LFP recordings and statistics

Spontaneous and odor-evoked spike and LFP recordings were initiated at least 1 week after implantation surgery, when the mice had recovered. Awake mice were head-fixed and were able to maneuver on an air-supported free-floating Styrofoam ball (Thinkerbiotech, Nanjing, China). An odor-port was positioned in front of the nose for odor delivery. Eight different odorants (isoamyl acetate, 2-heptanone, phenyl acetate, benzaldehyde, dimethylbutyric acid, n-heptane acid, n-pentanol, 2-pentanone, all purchased from Sinopharm Chemical Reagent Co.) were dissolved in mineral oil at 1% v/v dilution. Each odorant was presented for a total of 15 trials. All odors were presented for 2 s in a pseudorandom order, followed by an inter-stimulus interval of 20 s. For all the eight odors, except benzaldehyde, which is an aversive odor, the other seven odors are all neutral odors. We selected these odors since they usually evoke the strong responses in the electrophysiological recordings and they have been commonly used in our previous studies^27,60–62^.

For spike recordings, signals from the microelectrodes were sent to a headstage, amplified by a 16-channel amplifier; band-pass filtered at 300–5000 Hz, 2000× gain), and sampled at 40 kHz by an electrophysiological recording system (NeuroLego System, Jiangsu Brain Medical Technology Co. Ltd). Spikes were sorted from the raw data with the Offline Sorter V4 software (Plexon). Separation of different units was performed by principal component analysis. A unit was classified as a single unit if <0.75% of the interspike intervals were <1 ms, as described in our previous studies^56,58,61^. To generate the peristimulus time histograms, spikes 4 s before and 6 s after the onset of odor stimulation were extracted for each trial and the spike firing rate was averaged over 100-ms bins (Fig. 3). The average spike frequency during the 2 s before odor stimulation was calculated and defined as the spontaneous firing rate (baseline). The odor-evoked firing rate was calculated by averaging the frequency of spikes during the 2 s after the onset of odor stimulation. To test whether an odor evoked a significant response, the baseline firing rate and the odor-evoked firing rate across all the trials for each cell–odor pair were compared with a paired *t* test. If the *p* value was >0.05, the cell–odor pair was defined as nonresponsive. However, if the *p* value was <0.05, the cell–odor pair was defined as responsive. Responses were further categorized as excitatory (if the odor-evoked firing rate was higher than the baseline firing rate) or inhibitory (if the odor-evoked firing rate was lower than the baseline firing rate) (Fig. 3).

For LFP recordings, LFP signals were amplified (2000× gain, Plexon DigiAmp), filtered at 0.1–300 Hz, and sampled at 1 kHz. Spikes and LFP signals together with odor stimulation event markers were recorded via the same NeuroLego recording system. For LFP data processing, raw data 4 s prior to the onset of odor stimulation were selected as representing ongoing LFP activity. Data were filtered digitally with a Butterworth filter from 4 to 100 Hz using the MATLAB. Similar to previous studies, the theta band was filtered at 4–12 Hz, the beta band at 15–35 Hz, the low gamma band at 36–65 Hz, and the high-gamma band at 65–95 Hz, and LFP signals were averaged across each block. We focused on the beta and high-gamma bands in our analysis since odors usually evoke strong and reliable responses within these two frequency bands. To analyze odor-elicited changes in LFP power, we analyzed windows 5 s prior to and 10 s after the onset of odor stimulation. For each trial, the baseline was normalized to 1, and all trials for each odor stimulation were averaged with respect to the normalized data.

### Slice preparation and electrophysiology

Mice were deeply anesthetized with pentobarbital sodium and decapitated. The entire brain was quickly immersed in ice-cold sucrose-rich slicing solution (composition in mmol/l: 85 NaCl, 2.5 KCl, 4 MgCl_2_, 0.5 CaCl_2_, 1.25 NaH_2_PO_4_, 25 NaHCO_3_, 25 glucose, and 75 sucrose), equilibrated with 95% O_2_ and 5% CO_2_. Horizontal slices (300 μm) containing the OB were cut on a vibratome (Leica Biosystems, VT 1000S, Germany) and incubated at 37 °C in a chamber with oxygenated artificial cerebrospinal fluid (ACSF; composition in mmol/l: 119 NaCl, 2.5 KCl, 1.3 MgCl_2_, 2.5 CaCl_2_, 1.25 NaH_2_PO_4_, 1.3 NaHCO_3_, and 10 glucose) for 1 h and then maintained at room temperature (26 °C) for at least 1 h before being transferred to the recording chamber.

Electrophysiological experiments were performed as described previously^58^. OB slices were visualized with a ×60 objective on an upright microscope (ECLIPSE FN1, Nikon) equipped with wide-field fluorescence to identify fluorescently labeled neurons. The GFP-expressing mitral cells or GCs were recorded by patch clamp with a Multiclamp 700B amplifier (Molecular Devices). Mitral cells and GCs were identified by cell body morphology, location, and electrophysiological properties.

In current-clamp mode, recording pipettes were pulled from borosilicate glass capillaries (Sutter) to have resistances of 6–8 MΩ and then filled with ACSF for AP recording in cell-attached mode. For AP recording in whole-cell mode, pipettes were filled with a solution containing, in mM: 140 K-methylsulfate, 4 NaCl, 10 *N*-(2-hydroxyethyl) piperazine-N0-(2-ethanesulfonic acid), 0.2 ethylene glycol-bis(2-aminoethylether)-*N*, *N*, N0, N0-tetraacetic acid, 4 MgATP, 0.3 Na_3_GTP, and 10 phosphocreatine (pH adjusted to 7.4 with KOH).

To evoke AP firing, step currents (50, 100, 150, 200, 250, and 300 pA for mitral cells and 10, 20, 30, 40, and 50 pA for GCs, 400 ms duration) were injected into cells with an intertrial interval of 30 s.

To record olfactory-nerve-evoked responses, monophasic square pulses (200 μs, 400 μA) were delivered through a concentric electrode that was placed on the outermost layer of the OB, near the recorded mitral cell.

In voltage-clamp mode, mIPSCs were recorded at −65 mV holding potential while the slices were bathed in ACSF perfusion media including TTX (1 mM). The pipettes were filled with a CsCl-base internal solution containing (in mM): 135 CsCl, 10 *N*-(2-hydroxyethyl) piperazine-N0-(2-ethanesulfonic acid), 0.2 ethylene glycol-bis(2-aminoethylether)-*N*, *N*, N0, N0-tetraacetic acid, 2 Na_2_ATP, 0.3 Na_3_GTP, and 10 glucose.

For eIPSCs and PPD recordings, mice were injected with AAV-CMV-hm-Syn-ires-GFP or AAV-CMV-GFP mixed with AAV-VGAT-hChR2 (H134R)-mCherry-WPRE-hGH pA (2.05 × 10^12^ vg/ml) in the granular cell layer 3 weeks prior to recording. During recording, the cell membrane potential was held at −65 mV and photostimulation (473 nm, 20 mW) was delivered at an inter-stimulus interval of 80 ms through an optical fiber (200 μm OD, 0.22 NA; NEWDOON, Hangzhou, China) placed near the slice and connected to an intelligent optogenetic system (NEWDOON, Aurora-220, Hangzhou, China). The PPD was calculated as the peak current response to the second pulse divided by the peak current response to the first pulse.

### Western blotting

In order to examine the total α-synuclein and Ser129-phosphorylated α-synuclein levels in the OB, western blot analyses were carried out. Mice were sacrificed and their brains were removed. The OBs were dissected with a surgical blade and homogenized in lysis buffer with complete protease inhibitors and phosphatase inhibitors on ice. Tissue homogenates were collected and protein quantitation was performed by the Lowry method. Equal protein amounts of denatured samples were subjected to sodium dodecyl sulfate-polyacrylamide gel electrophoresis on 12% polyacrylamide gels and then transferred to nitrocellulose membranes. The immunoblots were incubated in 3% bovine serum albumin at room temperature for 3 h before being probed with primary (overnight at 4 °C) and appropriate secondary antibodies. The primary antibodies used were as follows: rabbit anti-α-synuclein (1:2000, cat. no. EPR20535; Abcam, Cambridge, MA), rabbit anti-pSer129 α-synuclein (1:1000, cat. no. EP1536Y; Abcam, Cambridge, MA), and mouse anti-β-actin (1:5000, cat. no. 66009-1-Ig; Proteintech, Inc.). Proteins were visualized using the enhanced chemiluminescence detection method. The scanned images were quantified with the ImageJ software. Specific bands were then quantified and normalized to the β-actin loading control for each lane and each blot.

### Preparation of brain tissue and immunofluorescence

Mice were intracardially perfused with cold saline followed by 4% paraformaldehyde (PFA, in 0.1 M phosphate-buffered saline (PBS); pH 7.4) and then brains were collected and postfixed for 6 h in 4% PFA at 4 °C. Brains were sectioned with a vibratome or freezing microtome at 30 μm in the coronal plane and blocked in 0.1 M PBS containing 10% serum and 0.3% Triton X-100 (Sangon Biotech) for 2 h at room temperature. Sections were treated with 10 μg/ml proteinase K (VICMED, diluted in Tris HCl buffer containing 10 mM Tris-HCl and 100 mM NaCl, pH 7.8). Sections were then incubated with anti-pSer129 α-synuclein (1:2000, diluted in 0.1 M PBS containing 1% serum and 0.1% Triton X-100) overnight at 4 °C, followed by incubation with appropriate secondary antibodies for 1 h and 4,6-diamidino-2-phenylindole (DAPI; nuclear stain; KeyGEN BioTECH, KGA215-10) for 10 min at room temperature. Labeled sections were imaged on a confocal scanning microscope (Zeiss, LSM710). The density of pSer129 α-synuclein-positive signals in different layers was analyzed using the ZEN Blue 3.0 software.

### TUNEL staining

TUNEL assays were performed by the In Situ Cell Death Detection Kit, TMR red (Roche). Briefly, the frozen sections were rinsed twice with PBS and permeabilized in 0.1% Triton X-100 on ice for 2 min. After being washed with PBS, the sections were incubated in the prepared TUNEL reaction mixture in a dark humid chamber for 60 min. Then the sections were rinsed with PBS again and sealed with DAPI. For the negative control, the section was incubated with label solution instead of TUNEL reaction mixture. For the positive control, the section was treated with DNase I before incubated with TUNEL reaction mixture.

### Fiber photometry

A fiber photometry system (Thinkertech, Nanjing, China) was used to record calcium signals from GCs, as described previously. Briefly, a 600 nl mixture of AAV virus (CMV-hm-Syn-ires-GFP or CMV-GFP; VGAT-Cre (2.60 × 10^12^ vg/ml); DIO-NES-jRGECO1a-WPRE-hGH pA (5.11 × 10^12^ vg/ml) = 1:0.8:1) was injected into the GCL (AP, 4.28 mm; ML, 1.0 mm; DV, 1.75 mm) of the right OB and then an optical fiber (230 μm OD, 0.37 NA; Thorlabs) was implanted 0.2 mm above the virus injection site. Spontaneous and odor-evoked calcium responses were recorded 3 weeks after surgery. The odor delivery method was as described above. To record the fluorescence signals, an LED (572 nm; Lumileds) was reflected by a dichroic mirror (67083; Edmund optics) and focused through an objective lens (×20, NA = 0.4; Olympus). An optical fiber (200 μm OD, 0.37 NA) guided the light between the commutator and the implanted optical fiber. The laser power was adjusted at the tip of the optical fiber to 40–60 μW. Fluorescence emission was band-pass filtered (87753, Edmund Optics) and detected by a photomultiplier tube (H10721, Hamamatsu). An amplifier was used to convert the photomultiplier tube current output to voltage, which was further filtered through a low-pass filter (35 Hz cut-off; Thinkertech). The analog voltage signals were digitalized at 100 Hz and recorded by the fiber photometry software (Thinkertech, Nanjing, China). Data were segmented on the basis of the onset of odor stimulation within individual trials. We derived the values of fluorescence change (Δ*F*/*F*) by calculating (*F* − *F*_0_)/*F*_0_, where *F*_0_ is the baseline fluorescence signal averaged over a 5-s long time window that preceded the onset of odor stimulation. To eliminate the varies of initial fluorescence signal value, we set it to the same value among different mice (in present study, we set the baseline to 1.75).

### Transmission electron microscopy

Mice were deeply anesthetized with pentobarbital sodium and intracardially perfused with ice-cold saline followed by 4% paraformaldehyde in 0.1 M phosphate buffer (pH 7.4). The OBs were dissected and cut into small blocks (1 mm × 1 mm × 1 mm), which were rapidly fixed in glutaraldehyde at 4 °C overnight. After washing 3 times in PBS, the tissue blocks were fixed in 1% osmium tetroxide, stained with a 2% aqueous solution of uranyl acetate, and then dehydrated with a concentration gradient sequence of ethanol and acetone. Finally, the tissue blocks were embedded in epoxy resin. Ultrathin sections (70 nm) were cut with an ultramicrotome, collected on copper grids, counter-stained with 4% uranyl acetate and lead citrate, and then observed under a Tecnai G2 Spirit TWIN electron microscope (FEI). The presynaptic membrane area and mitochondrial aspect ratio were measured with the ImageJ software (v1.8.0, NIH).

### Golgi staining

A rapid Golgi staining kit (GolgiStain Kit, FD Neurotechnologies) was used to stain the GCs in the OB. In brief, mice brains were collected and stored in the dark for 21 days in the Golgi-Cox solution before being transferred to a cryoprotection solution for 1 week in the dark and sectioned coronally at a thickness of 100 μm with a vibratome. The sections were collected on clean gelatin-coated microscope slides and then stained with the staining solution. After rinsing in distilled water and dehydrated in successive baths of alcohol, sections were cleared in a xylene solution. Sections were mounted on glass coverslips using a neutral resinous medium and scanned under a Slice Scanner (VS120, Olympus). ImageJ (v1.8.0, NIH) was used to analyze the differences between-groups for dendritic and spine morphology by observers blinded to the experimental conditions. For the total dendritic length of GCs, we measured the visible dendrites in one image. Spines on some of the secondary dendrites of GCs were counted and the length of the dendrite was measured. Spine density was represented as the number of spines/10 μm of dendritic length. The number of cells used in each analysis is indicated in the figure legend.

### Statistics

All statistical analyses were conducted in MATLAB 2020a and GraphPad Prism 7.0. Data throughout are presented as mean ± SEM. All morphological, behavioral, electrophysiological, and biochemical data were obtained by counterbalancing experimental conditions with controls. The Gaussian distribution of the data was assessed before conducting statistics. If the data sets were normally distributed, *t* test was used (two unrelated samples) or two-way ANOVA (>2 unrelated samples); if the data was not a normal distribution, Mann–Whitney test (two unrelated samples), Wilcoxon signed-rank test (two related samples), or the K-W test (>2 unrelated samples) should be used. Tukey post hoc tests were used to directly assess group differences following ANOVA where appropriate. The data analyses and statistics are described in the “Results“ section where these data are mentioned.

### Reporting summary

Further information on research design is available in the Nature Research Reporting Summary linked to this article.

## Supplementary information

Reporting Summary

Supplementary Information

## Data Availability

The data acquired and analyzed for this study are available from the corresponding authors upon reasonable request.

## Code availability

All the MATLAB codes is available at https://github.com/liekkas121/Synuclein-aggregation-in-the-olfactory-bulb.

## References

[1] Doty RL (2012). Olfactory dysfunction in Parkinson disease. Nat. Rev. Neurol..

[2] Fullard ME, Morley JF, Duda JE (2017). Olfactory dysfunction as an early biomarker in Parkinson’s disease. Neurosci. Bull..

[3] Doty RL, Deems DA, Stellar S (1988). Olfactory dysfunction in parkinsonism: a general deficit unrelated to neurologic signs, disease stage, or disease duration. Neurology.

[4] Niu H (2018). Alpha-synuclein overexpression in the olfactory bulb initiates prodromal symptoms and pathology of Parkinson’s disease. Transl. Neurodegener..

[5] Wang QJ (2020). Noncanonical roles of halpha-syn (A53T) in the pathogenesis of Parkinson’s disease: synaptic pathology and neuronal aging. Neural Plast..

[6] Fredenburg RA (2007). The impact of the E46K mutation on the properties of alpha-synuclein in its monomeric and oligomeric states. Biochemistry.

[7] Braak H, Ghebremedhin E, Rüb U, Bratzke H, Del Tredici K (2004). Stages in the development of Parkinson’s disease-related pathology. Cell Tissue Res..

[8] Crespo Cuevas AM (2018). Distinctive olfactory pattern in Parkinson’s disease and non-neurodegenerative causes of hyposmia. Neurodegener. Dis..

[9] Rey NL, Wesson DW, Brundin P (2018). The olfactory bulb as the entry site for prion-like propagation in neurodegenerative diseases. Neurobiol. Dis..

[10] Li A, Rao X, Zhou Y, Restrepo D (2020). Complex neural representation of odour information in the olfactory bulb. Acta Physiol..

[11] Farrell KF (2014). Non-motor parkinsonian pathology in aging A53T alpha-synuclein mice is associated with progressive synucleinopathy and altered enzymatic function. J. Neurochem..

[12] Petit GH (2013). Rasagiline ameliorates olfactory deficits in an alpha-synuclein mouse model of Parkinson’s disease. PLoS ONE.

[13] Zhang S, Xiao Q, Le W (2015). Olfactory dysfunction and neurotransmitter disturbance in olfactory bulb of transgenic mice expressing human A53T mutant alpha-synuclein. PLoS ONE.

[14] Rey NL (2016). Widespread transneuronal propagation of α-synucleinopathy triggered in olfactory bulb mimics prodromal Parkinson’s disease. J. Exp. Med..

[15] Zhang XM (2019). The A30P alpha-synuclein mutation decreases subventricular zone proliferation. Hum. Mol. Genet..

[16] Neuner J, Filser S, Michalakis S, Biel M, Herms J (2014). A30P alpha-Synuclein interferes with the stable integration of adult-born neurons into the olfactory network. Sci. Rep..

[17] Neuner J (2014). Pathological alpha-synuclein impairs adult-born granule cell development and functional integration in the olfactory bulb. Nat. Commun..

[18] Taguchi K, Watanabe Y, Tsujimura A, Tanaka M (2020). alpha-Synuclein promotes maturation of immature juxtaglomerular neurons in the mouse olfactory bulb. Mol. Neurobiol..

[19] Kulkarni AS (2020). Perturbation of in vivo neural activity following α-synuclein seeding in the olfactory bulb. J. Parkinsons Dis..

[20] Fleming SM (2008). Olfactory deficits in mice overexpressing human wildtype alpha-synuclein. Eur. J. Neurosci..

[21] Valle-Leija P, Drucker-Colín R (2014). Unilateral olfactory deficit in a hemiparkinson’s disease mouse model. Neuroreport.

[22] Talaga AK, Dong FN, Reisert J, Zhao H (2017). Cilia- and flagella-associated protein 69 regulates olfactory transduction kinetics in mice. J. Neurosci..

[23] Zhang W (2019). Partial depletion of dopaminergic neurons in the substantia nigra impairs olfaction and alters neural activity in the olfactory bulb. Sci. Rep..

[24] Kobayakawa K (2007). Innate versus learned odour processing in the mouse olfactory bulb. Nature.

[25] Saraiva LR (2016). Combinatorial effects of odorants on mouse behavior. Proc. Natl Acad. Sci. USA.

[26] Kay LM, Laurent G (1999). Odor- and context-dependent modulation of mitral cell activity in behaving rats. Nat. Neurosci..

[27] Doucette W (2011). Associative cortex features in the first olfactory brain relay station. Neuron.

[28] Ishiguro M, Kobayashi S, Matsuyama K, Nagamine T (2019). Effects of propofol on IPSCs in CA1 and dentate gyrus cells of rat hippocampus: propofol effects on hippocampal cells’ IPSCs. Neurosci. Res..

[29] Ivanova SY, Storozhuk MV, Kostyuk PG (2002). Changes in paired pulse depression as an indicator for the involvement of presynaptic mechanism(s) in modulation of GABA-ergic synaptic transmission in rat hippocampal cell cultures. Neurophysiology.

[30] Yazawa I (2005). Mouse model of multiple system atrophy alpha-synuclein expression in oligodendrocytes causes glial and neuronal degeneration. Neuron.

[31] Taguchi T (2020). alpha-Synuclein BAC transgenic mice exhibit RBD-like behaviour and hyposmia: a prodromal Parkinson’s disease model. Brain.

[32] Saito Y (2016). Lewy body pathology involves the olfactory cells in Parkinson’s disease and related disorders. Mov. Disord..

[33] Vaaga CE, Westbrook GL (2016). Parallel processing of afferent olfactory sensory information. J. Physiol..

[34] Busche MA (2008). Clusters of hyperactive neurons near amyloid plaques in a mouse model of Alzheimer’s disease. Science.

[35] Palop JJ (2007). Aberrant excitatory neuronal activity and compensatory remodeling of inhibitory hippocampal circuits in mouse models of Alzheimer’s disease. Neuron.

[36] Hu B, Geng C, Hou XY (2017). Oligomeric amyloid-beta peptide disrupts olfactory information output by impairment of local inhibitory circuits in rat olfactory bulb. Neurobiol. Aging.

[37] Wesson DW (2011). Sensory network dysfunction, behavioral impairments, and their reversibility in an Alzheimer’s β-amyloidosis mouse model. J. Neurosci..

[38] Rosenberg A, Patterson JS, Angelaki DE (2015). A computational perspective on autism. Proc. Natl Acad. Sci. USA.

[39] Hu B (2021). GABA(A) receptor agonist muscimol rescues inhibitory microcircuit defects in the olfactory bulb and improves olfactory function in APP/PS1 transgenic mice. Neurobiol. Aging.

[40] Kato HK, Gillet SN, Peters AJ, Isaacson JS, Komiyama T (2013). Parvalbumin-expressing interneurons linearly control olfactory bulb output. Neuron.

[41] Ito H, Nakayama K, Jin C, Suzuki Y, Yazawa I (2012). alpha-Synuclein accumulation reduces GABAergic inhibitory transmission in a model of multiple system atrophy. Biochem. Biophys. Res. Commun..

[42] Danzer KM (2007). Different species of alpha-synuclein oligomers induce calcium influx and seeding. J. Neurosci..

[43] Ludtmann MHR (2018). alpha-synuclein oligomers interact with ATP synthase and open the permeability transition pore in Parkinson’s disease. Nat. Commun..

[44] Hsu LJ (2000). alpha-synuclein promotes mitochondrial deficit and oxidative stress. Am. J. Pathol..

[45] Prots I (2018). alpha-Synuclein oligomers induce early axonal dysfunction in human iPSC-based models of synucleinopathies. Proc. Natl Acad. Sci. USA.

[46] Hashimoto M (2004). The role of alpha-synuclein assembly and metabolism in the pathogenesis of Lewy body disease. J. Mol. Neurosci..

[47] Alim MA (2004). Demonstration of a role for alpha-synuclein as a functional microtubule-associated protein. J. Alzheimers Dis..

[48] Scott DA (2010). A pathologic cascade leading to synaptic dysfunction in alpha-synuclein-induced neurodegeneration. J. Neurosci..

[49] Volpicelli-Daley LA (2011). Exogenous alpha-synuclein fibrils induce Lewy body pathology leading to synaptic dysfunction and neuron death. Neuron.

[50] Yamamoto K, Izumi Y, Arifuku M, Kume T, Sawada H (2019). alpha-Synuclein oligomers mediate the aberrant form of spike-induced calcium release from IP3 receptor. Sci. Rep..

[51] De la Rosa-Prieto C, Saiz-Sanchez D, Ubeda-Banon I, Flores-Cuadrado A, Martinez-Marcos A (2016). Neurogenesis, neurodegeneration, interneuron vulnerability, and amyloid-β in the olfactory bulb of APP/PS1 mouse model of Alzheimer’s disease. Front. Neurosci..

[52] Linster C, Cleland TA (2016). Neuromodulation of olfactory transformations. Curr. Opin. Neurobiol..

[53] Fletcher ML, Chen WR (2010). Neural correlates of olfactory learning: critical role of centrifugal neuromodulation. Learn. Mem..

[54] Padmanabhan K (2016). Diverse representations of olfactory information in centrifugal feedback projections. J. Neurosci..

[55] Hoglinger GU (2015). A new dopaminergic nigro-olfactory projection. Acta Neuropathol..

[56] Wu, J. et al. Excitability of neural activity is enhanced, but neural discrimination of odors is slightly decreased, in the olfactory bulb of fasted mice. *Genes*10.3390/genes11040433. (2020). 10.3390/genes11040433 PMC7230403 32316323

[57] Muthusamy N, Zhang X, Johnson CA, Yadav PN, Ghashghaei HT (2017). Developmentally defined forebrain circuits regulate appetitive and aversive olfactory learning. Nat. Neurosci..

[58] Sun C (2019). Leptin modulates olfactory discrimination and neural activity in the olfactory bulb. Acta Physiol..

[59] Li A, Gire DH, Restrepo D (2015). ϒ spike-field coherence in a population of olfactory bulb neurons differentiates between odors irrespective of associated outcome. J. Neurosci..

[60] Li A, Guthman EM, Doucette WT, Restrepo D (2017). Behavioral status influences the dependence of odorant-induced change in firing on prestimulus firing rate. J. Neurosci..

[61] Wang D (2019). Task-demand-dependent neural representation of odor information in the olfactory bulb and posterior piriform cortex. J. Neurosci..

[62] Sun C (2021). Oxytocin modulates neural processing of mitral/tufted cells in the olfactory bulb. Acta Physiol..

