# Supplementary material for: α-Synuclein aggregation in the olfactory bulb induces olfactory deficits by perturbing granule cells and granular–mitral synaptic transmission
Source: NPJ Parkinsons Dis. 2021 Dec 13;7:114. doi: 10.1038/s41531-021-00259-7 (PMC8668919; doi:10.1038/s41531-021-00259-7)
Supplement: Supplementary file 1 — Reporting Summary [file 41531_2021_259_MOESM1_ESM.pdf]

nature portfolio

Corresponding author(s): DBPR NPJPARKD-00740R1

Last updated by author(s): Oct 30, 2021

Reporting Summary

Nature Portfolio wishes to improve the reproducibility of the work that we publish. This form provides structure for consistency and transparency in reporting. For further information on Nature Portfolio policies, see our Editorial Policies and the Editorial Policy Checklist.

Statistics

For all statistical analyses, confirm that the following items are present in the figure legend, table legend, main text, or Methods section.

n/a	Confirmed	
☐	☒	The exact sample size (*n*) for each experimental group/condition, given as a discrete number and unit of measurement
☐	☒	A statement on whether measurements were taken from distinct samples or whether the same sample was measured repeatedly
☐	☒	The statistical test(s) used AND whether they are one- or two-sided<br>*Only common tests should be described solely by name; describe more complex techniques in the Methods section.*
☐	☒	A description of all covariates tested
☐	☒	A description of any assumptions or corrections, such as tests of normality and adjustment for multiple comparisons
☐	☒	A full description of the statistical parameters including central tendency (e.g. means) or other basic estimates (e.g. regression coefficient) AND variation (e.g. standard deviation) or associated estimates of uncertainty (e.g. confidence intervals)
☐	☒	For null hypothesis testing, the test statistic (e.g. *F*, *t*, *r*) with confidence intervals, effect sizes, degrees of freedom and *P* value noted<br>*Give P values as exact values whenever suitable.*
☒	☐	For Bayesian analysis, information on the choice of priors and Markov chain Monte Carlo settings
☒	☐	For hierarchical and complex designs, identification of the appropriate level for tests and full reporting of outcomes
☐	☒	Estimates of effect sizes (e.g. Cohen's *d*, Pearson's *r*), indicating how they were calculated

Our web collection on statistics for biologists contains articles on many of the points above.

Software and code

Policy information about availability of computer code

Data collection	We listed all softwares used in the experiments and for analysis in the Methods section. We used an electrophysiological recording system (NeuroLego System, Jiangsu Brain Medical Technology Co. Ltd) to collect Spikes and LFP signals. We used the Multiclamp 700B amplifier (Molecular Devices) and Digidata 1440A interface (Molecular Devices) to record the in-vitro electrophysiological data. We used a fiber photometry system (Thinkertech, Nanjing, China) to record calcium signals. Confocal images were captured with Zen program from LSM710 (Zeiss). Transmission electron microscopy images were captured under a Tecnai G2 Spirit TWIN electron microscope (FEI). Golgi staining images were scanned under a Slice Scanner (VS120, Olympus).
Data analysis	We listed all softwares used in the experiments and for analysis in the Methods section. The raw spikes data were sorted with Offline Sorter V4 software (Plexon), the LFP raw data were filtered and then analyzed using customized code written in MATLAB. Confocal images data and other image data were analyzed using ZEN Blue 3.0 software and ImageJ (v1.8.0, NIH) with custom settings. All statistical analysis were performed using GraphPad Prism 7.0 and MATLAB 2020a.

For manuscripts utilizing custom algorithms or software that are central to the research but not yet described in published literature, software must be made available to editors and reviewers. We strongly encourage code deposition in a community repository (e.g. GitHub). See the Nature Portfolio guidelines for submitting code & software for further information.

Data

Policy information about availability of data

All manuscripts must include a data availability statement. This statement should provide the following information, where applicable:

- Accession codes, unique identifiers, or web links for publicly available datasets
- A description of any restrictions on data availability
- For clinical datasets or third party data, please ensure that the statement adheres to our policy

The data that support the findings in this study are available from the corresponding authors upon reasonable request. All codes described in the study are available at https://github.com/liekkas121/Synuclein-aggregation-in-the-olfactory-bulb.

Field-specific reporting

Please select the one below that is the best fit for your research. If you are not sure, read the appropriate sections before making your selection.

☒ Life sciences ☐ Behavioural & social sciences ☐ Ecological, evolutionary & environmental sciences

For a reference copy of the document with all sections, see nature.com/documents/nr-reporting-summary-flat.pdf

Life sciences study design

All studies must disclose on these points even when the disclosure is negative.

Sample size	No statistical methods were used to pre-determine sample sizes but our sample sizes in the data are similar with previous works in this field.
Data exclusions	The data from mice without correct virus expression pattern were excluded. Data sets in this study were analyzed using GraphPad Prism 7.0 to exclude the outliers.
Replication	Experimental findings were reliably reproduced among all subjects in all experiments comprised of multiple cohorts.
Randomization	For each experiment in this study, mice were randomly selected as control group and experimental group.
Blinding	During image analysis, the experimenter was blind to the group information.

Reporting for specific materials, systems and methods

We require information from authors about some types of materials, experimental systems and methods used in many studies. Here, indicate whether each material, system or method listed is relevant to your study. If you are not sure if a list item applies to your research, read the appropriate section before selecting a response.

Materials & experimental systems

n/a	Involved in the study	
☐	☒	Antibodies
☒	☐	Eukaryotic cell lines
☒	☐	Palaeontology and archaeology
☐	☒	Animals and other organisms
☒	☐	Human research participants
☒	☐	Clinical data
☒	☐	Dual use research of concern

Methods

n/a	Involved in the study	
☒	☐	ChIP-seq
☒	☐	Flow cytometry
☒	☐	MRI-based neuroimaging

Antibodies

Antibodies used	We listed all antibodies used with their catalog number in Methods section. Primary antibodies: rabbit anti-α-synuclein (1:2000, cat. no. EPR20535; Abcam, Cambridge, MA), rabbit anti-pSer129 α-synuclein (1:1000, cat. no. EP1536Y; Abcam, Cambridge, MA), and mouse anti-β-actin (1:5000, cat. no. 66009-1-Ig; Proteintech, Inc).
Validation	https://www.abcam.cn/alpha-synuclein-antibody-epr20535-bsa-and-azide-free-ab225866.html<br>https://www.abcam.cn/alpha-synuclein-phospho-s129-antibody-ep1536y-ab51253.html<br>https://www.ptgcn.com/products/Pan-Actin-Antibody-66009-1-Ig.htm

Animals and other organisms

Policy information about studies involving animals; ARRIVE guidelines recommended for reporting animal research

Laboratory animals	We described the subjects in Methods section (Animals). Mice were housed, maintained, and used in experiments under the regulations, approval, and animal care standards of the Institutional Animal Care and Use Committee of Xuzhou Medical University. Adult male C57BL/6J mice were group housed on a reverse light cycle until they underwent surgery, after which mice were housed individually for at least one week for recovery before further experiments.
Wild animals	The study did not involve wild animals.
Field-collected samples	The study did not involve samples collected from the field.
Ethics oversight	All animal experiments were performed under the regulations, approval, and animal care standards of the Institutional Animal Care and Use Committee of Xuzhou Medical University. We complied with all pertinent ethical regulations.

Note that full information on the approval of the study protocol must also be provided in the manuscript.
