# Supplementary material for: α-Synuclein aggregation in the olfactory bulb induces olfactory deficits by perturbing granule cells and granular–mitral synaptic transmission
Source: NPJ Parkinsons Dis. 2021 Dec 13;7:114. doi: 10.1038/s41531-021-00259-7 (PMC8668919; doi:10.1038/s41531-021-00259-7)
Supplement: Supplementary file 2 — Supplementary Information [file 41531_2021_259_MOESM2_ESM.pdf]

α-Synuclein aggregation in the olfactory bulb induces olfactory deficits by perturbing granule cells and granular–mitral synaptic transmission

Fengjiao Chen[1,3], Wei Liu[1,3], Penglai Liu[1], Zhen Wang[2], You Zhou[2], Xingyu Liu[2], Anan Li[1,*]

[1]Jiangsu Key Laboratory of Brain Disease and Bioinformation, Research Center for Biochemistry and Molecular Biology, Xuzhou Medical University, Xuzhou, China

[2]School of Life Science, Xuzhou Medical University, Xuzhou, China

[3]These authors contributed equally.

Running title: Mechanism of α-synuclein aggregation-induced olfactory deficit

Corresponding author: Anan Li, Address: 209th Tongshan Road, Xuzhou, Jiangsu, China, 221004; Email: anan.li@xzhmu.edu.cn; Tel: 86-516-83262621

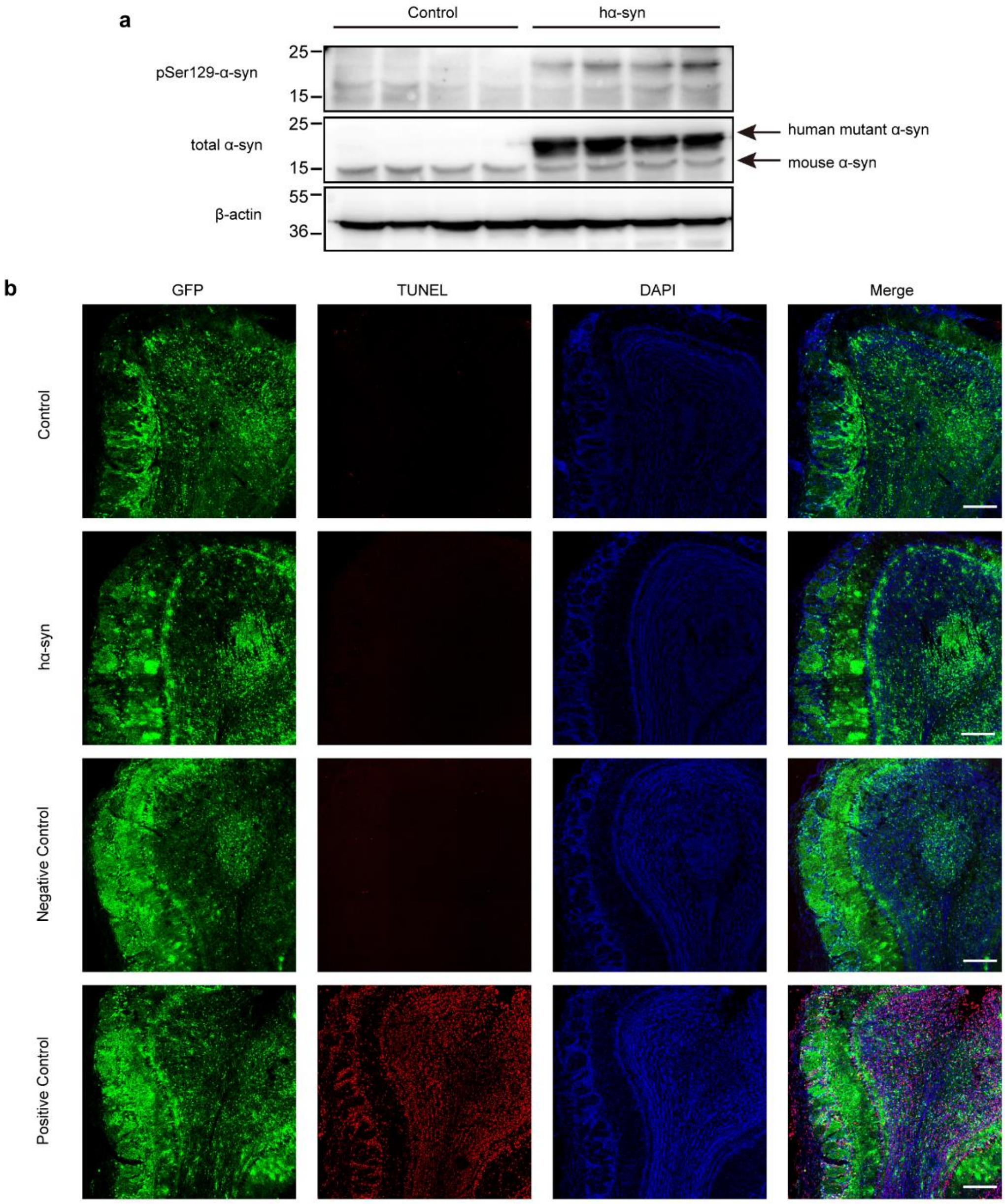

Supplementary Fig. 1 Exogenous human α-synuclein expression does not induce cell apoptosis in the OB. **a** The expression of pSer129-α-syn and total α-syn in OB tissue from all four mice of each group three weeks after viral injection. **b** The representative TUNEL staining images of OB from control and hα-syn mouse three weeks after viral injection. α-syn, α-synuclein. Scale bars = 200 μm.

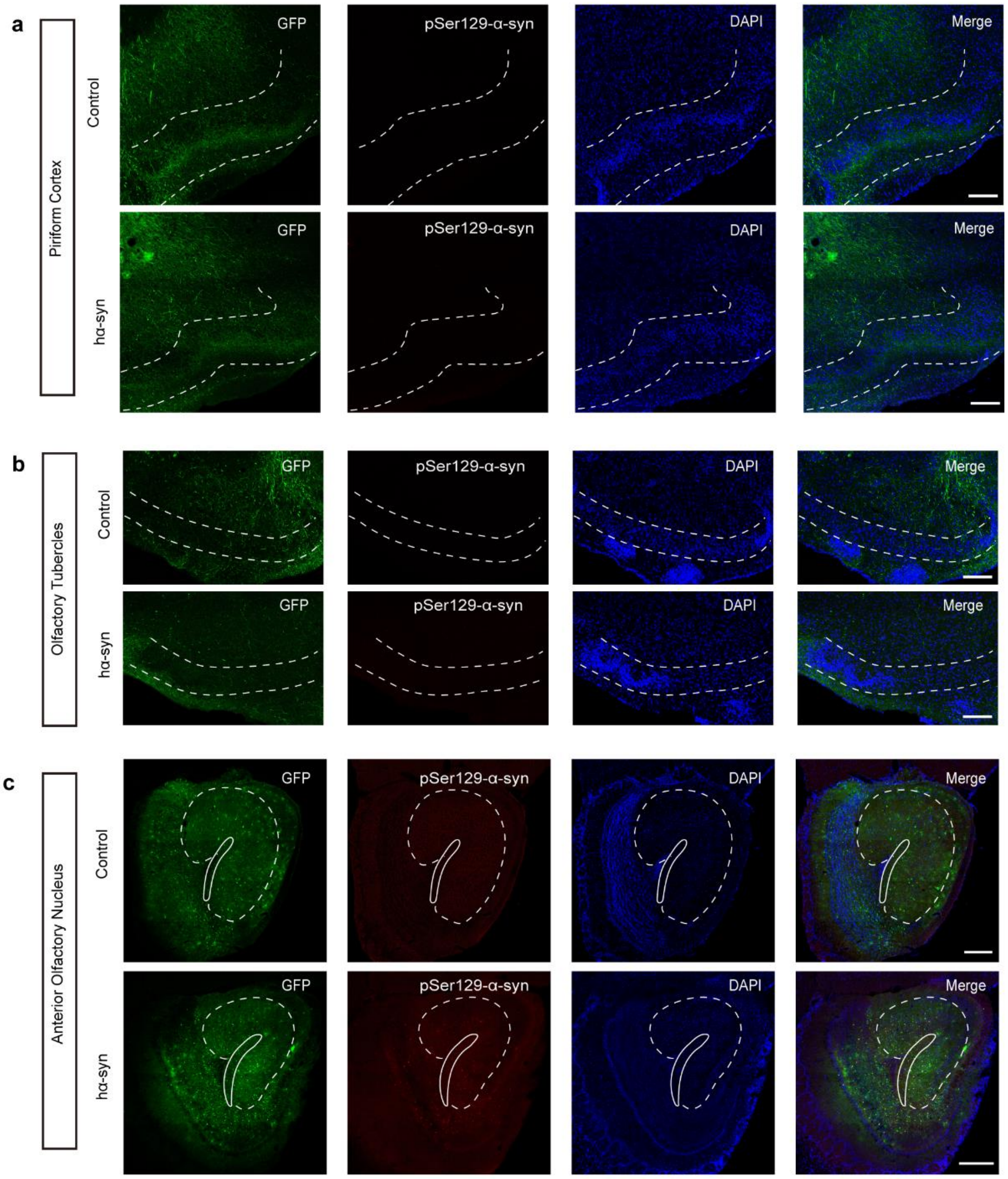

Supplementary Fig. 2 The α-synuclein pathology pattern in the higher olfactory areas. The representative images of piriform cortex (scale bars = 200 μm) (**a**), olfactory tubercles (scale bars = 200 μm) (**b**) and anterior olfactory nucleus (scale bars = 300 μm) (**c**) areas from each group immunostained with anti-pSer129-α-syn antibody three weeks after viral injection. The corresponding olfactory regions were circled by white dashed lines.

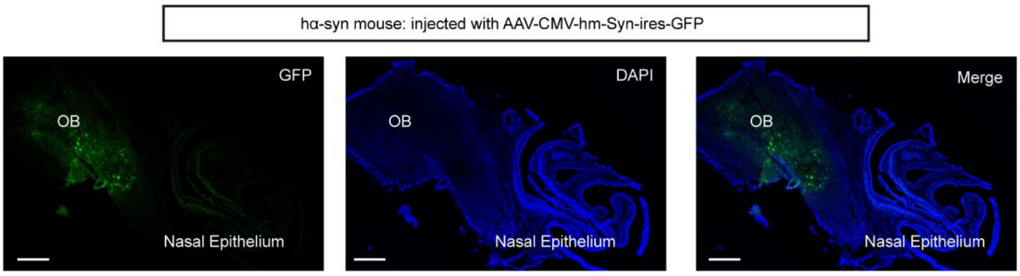

Supplementary Fig. 3 The virus expression pattern in OB and nasal epithelium. The representative images from hα-syn group show that there was no GFP^{+} cells in nasal epithelium three weeks after the viral injection. Scale bars = 500 μm.

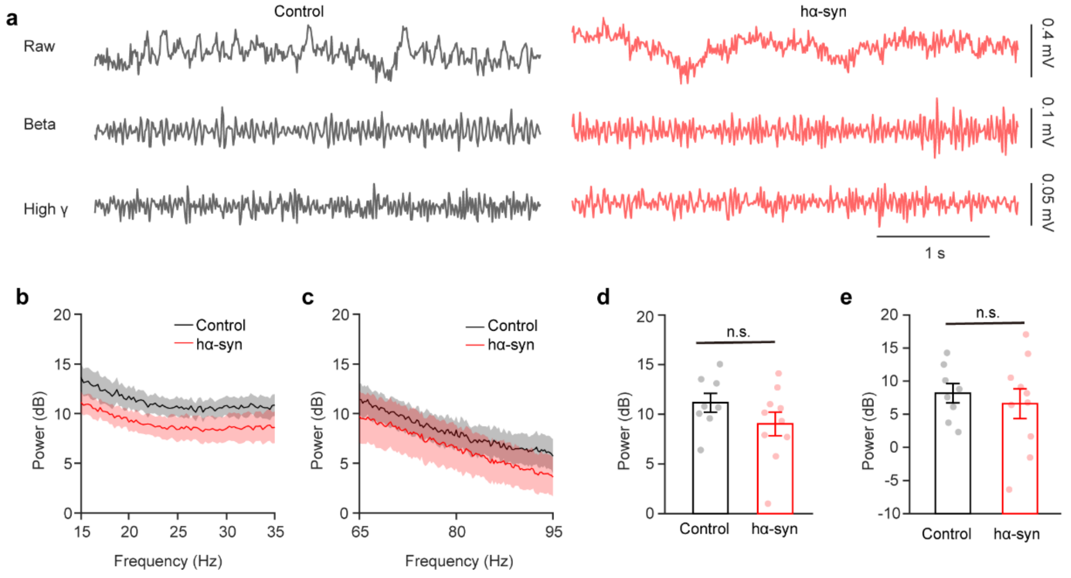

Supplementary Fig. 4 α-Synuclein aggregates have no significant effect on spontaneous LFP activity in the OB. **a** Raw traces (top) and filtered beta (middle) and high-gamma (bottom) oscillations in the LFP signals recorded from representative mice in the control and hα-syn groups. **b** and **c** The averaged power spectrum of the ongoing beta (**b**) and high-gamma (**c**) LFP signals across each group of mice recorded. **d** and **e** Statistical analysis of the difference in power in the beta (**d**) and high-gamma (**e**) bands between recordings from the control (n = 8) and hα-syn (n = 10) groups. n.s., not significant. Data are presented as the mean ± SEM.

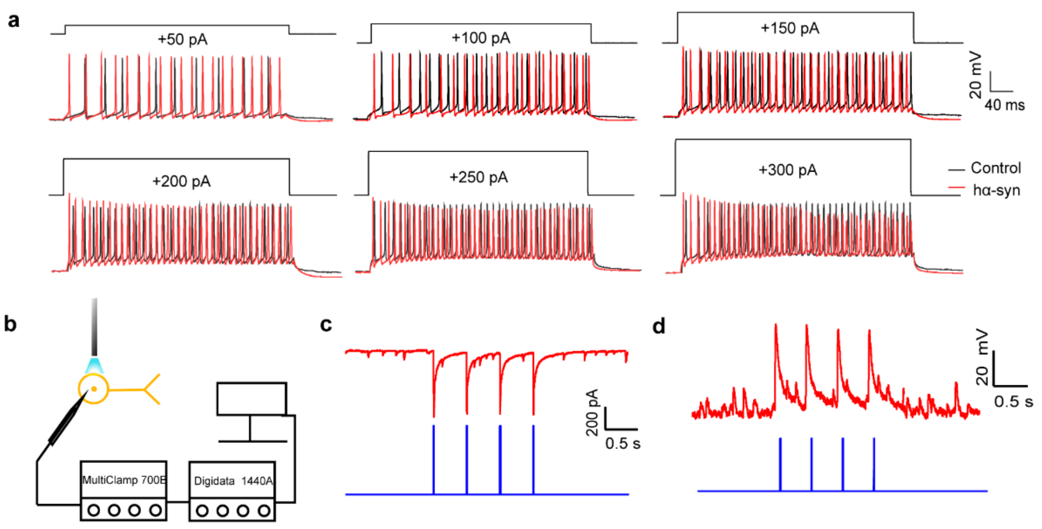

Supplementary Fig. 5 The representative data from patch-clamp recordings. **a** The representative current injection-evoked mitral cell APs recorded with the whole-cell patch-clamping mode. **b** Diagram of optogenetic activation of the granule cells. **c** and **d** The representative light-evoked currents (**c**) and APs (**d**) recorded from a granule cell.

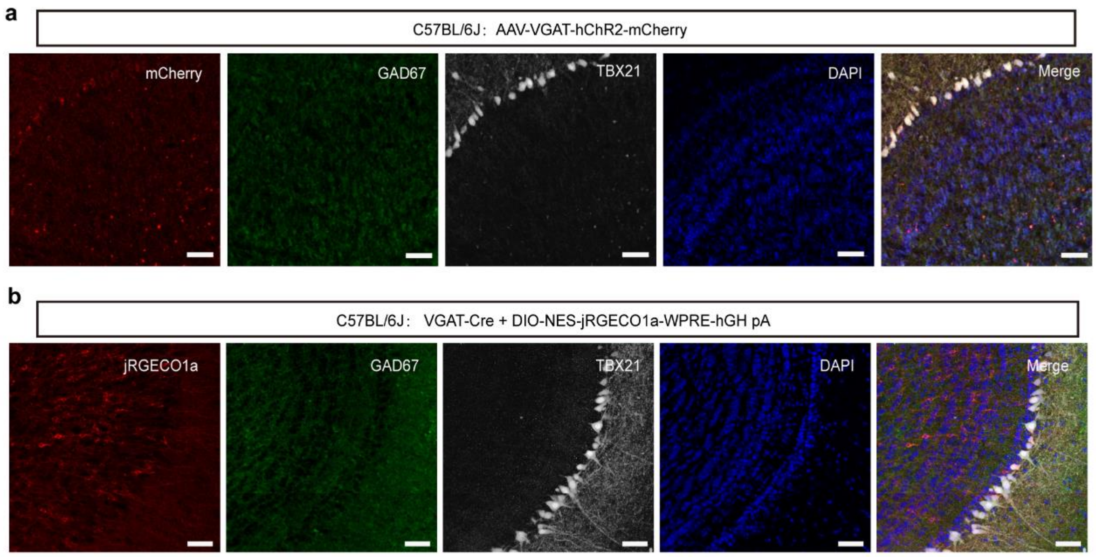

Supplementary Fig. 6 Verification of the expression specificity for the virus with VGAT promoter. The representative immunofluorescence images show that the hChR2-mCherry (**a**) and jRGECO1a (**b**) expressed mainly in granule cell layer. GAD67, a marker for GABAergic neurons; TBX21, a marker for M / T cells in OB. Scale bars = 50 μm.
